# The impact of reward scalarization and weight scheduling on optimization dynamics in multi-objective molecular design

**DOI:** 10.1186/s13321-026-01273-8

**Published:** 2026-08-19

**Authors:** Lucas Leuschner, Oscar Palomino-Hernandez

**Affiliations:** 1 Department of Chemistry, Johannes Gutenberg University Mainz, Duesbergweg 10-14, 55128 Mainz, Germany; 2 Institute for Quantitative and Computational Biosciences (IQCB), Johannes Gutenberg University Mainz, Johannes-von-Müller-Weg 6, 55128 Mainz, Germany

**Keywords:** De novo molecular generation, Reinforcement learning fine-tuning, Multi-objective optimization, Scalarization, Desirability functions, Molecular design

## Abstract

Inverse molecular design imposes multiple property constraints, requiring them to be combined into a scalar reward for reinforcement-learning (RL) fine-tuning; a step that can be highly sensitive to the reward formulation. Here, we systematically analyze how scalarization choices and stabilization mechanisms shape optimization dynamics in RL fine-tuning for multi-parameter optimization (MPO). We evaluate three scalarization schemes (arithmetic mean, geometric mean, and Chebyshev) under multiple target regimes that vary in constraint tightness and prior support. While broad targets yield stable behavior across scalarizations, narrow and weakly supported targets expose sharp failure modes. We further show that these failure modes can be prevented through the usage of target-aware weight scheduling. Collectively, our results highlight critical interactions between scalarization choice and weight dynamics, and provide mechanistic insight and actionable guidance for stabilizing RL fine-tuning in constrained molecular MPO.

**Scientific contribution**

This work examines different reward formulations in multi-objective reinforcement learning for molecular design, going beyond the designs typically considered in prior studies. We further show how these formulations influence convergence behavior and collapse modes, and how their effects depend on the size and constraint structure of the multi-dimensional optimization space.

**Supplementary Information:**

The online version contains supplementary material available at 10.1186/s13321-026-01273-8.

## Introduction

Inverse molecular design aims to generate novel chemical structures with desired properties for drug discovery and other applications [1, 2]. Traditional approaches often involve resource-intensive screening of vast chemical libraries or iterative chemical synthesis, processes that are both time-consuming and expensive. Moreover, the chemical space of drug-like molecules is vast (often estimated on the order of $10^{60}$ possible structures) [3], making exhaustive search infeasible and motivating data-driven approaches.

Computational methods offer a promising avenue to accelerate this process by designing entirely new molecules in silico [4–6]. Fragment-based structure generators, such as those employing chemically reasonable mutations (CReM), explicitly guarantee chemical validity and improve synthetic feasibility by swapping molecular fragments within equivalent local contexts [7]. Other approaches leverage evolutionary algorithms to optimize binding site complementarity [8], or fingerprint-based methods to reverse-engineer molecular structures from relevant bits [9].

On the line of deep generative algorithms, models based on Recurrent Neural Networks (RNNs) [10], Transformers [11, 12], and Graph Neural Networks (GNNs) [13] have shown remarkable success in learning distributions over chemical structures and generating syntactically valid molecules [5, 14]. However, steering these models towards specific, desirable regions of chemical space was challenging. Early work by Segler et al. addressed this by relying purely on maximum-likelihood estimation (MLE) training to bias models towards target properties, for example through filtered training sets or post-hoc selection of generated molecules [10]. More recently, reinforcement learning (RL) has become a widely adopted alternative for fine-tuning these generative models towards task-specific objectives, thereby overcoming the requirement for large datasets of molecules with the specific desired properties [15].

### RL-based fine-tuning

RL-guided fine-tuning typically requires an initial generative model, trained to reproduce the probability distribution of molecular sequences of a given dataset. During the fine-tuning process, this model is used initially to generate a molecular sequence *X*. This sequence is then evaluated by a reward function *R* encoding any desired feature score *f*. Thus, $R(X) = f(X) \in \mathbb {R}$. Using this reward signal, the model parameters are updated via policy-based or value-based RL methods to increase the likelihood of molecular sequences $X_n$ with higher reward, thereby biasing the underlying generation process towards molecules that better satisfy the target property.

The use of RL for fine-tuning molecular generators emerged around 2017, when several groups proposed closely related Prior+RL schemes for sequence models. Jaques et al. introduced Sequence Tutor as a framework for conservative fine-tuning of sequence models [16]. An RNN is first trained on an initial dataset and then kept frozen as a *Prior*. A separate RL learner (a value-based Deep Q-Network variant) is initialized from the pretrained weights and optimized to improve sequence quality, while a KL penalty constrains the learned policy to remain close to the Prior distribution. Subsequently, Olivecrona et al. [15] explored policy-gradient methods such as REINFORCE [17] for molecular generation. As in Sequence Tutor, the Prior is frozen and an *Agent* network is initialized from its weights and updated. Crucially, they implement the proximity constraint by explicitly coupling the Prior and Agent log-likelihoods within the learning signal (rather than treating KL as an external regularizer), which they found to yield the best performance. This idea later evolved into the REINVENT framework, which has become a *de facto* standard for RL-based next-token molecular generation [18]. These early contributions established the core recipe that most modern RL-based molecular design frameworks still follow: (i) pretrain a generative model as a Prior on large chemical datasets, (ii) define a scalar scoring function (reward) possibly aggregating multiple property predictors, and (iii) apply RL updates that optimize the model under the evaluated reward while regularizing the policy close to the Prior to avoid unrealistic out-of-distribution molecules.

### From single-objective to multi-objective rewards

In practice, a successful drug-like molecule must satisfy multiple property criteria simultaneously: high potency at the target, acceptable physicochemical and ADME (absorption, distribution, metabolism, excretion) profiles, among others [19, 20]. Balancing these objectives is a core challenge in multi-parameter optimization (MPO) paradigms [21, 22], as improving one property can potentially affect others (e.g. target affinity vs. solubility). In this multi-objective setting, a molecular sequence *X* is associated with a feature score vector $\textbf{f}(X) = (f_1(X), \dots , f_k(X))$, where each $f_i(X)$ measures these distinct property of interest. To use the RL fine-tuning framework described above, a scalarization function *S* is introduced to map this vector-valued feature score to a scalar reward. The resulting reward is given by $R(X) = S(\textbf{f}(X), \textbf{w})$, where $\textbf{w}$ is a weight vector defining user- or project-specific preferences.

This scalarization-based formulation allows both single-objective and multi-objective design problems to be treated with the same RL fine-tuning framework. However, this convenience comes at a cost. The choice of scalarization function (e.g. desirability transforms, bounds, and functional form) directly shapes the reward landscape, including whether the agent can effectively explore concave or more complex manifolds. The choice of weights (their relative values, and whether they are static or adaptively updated during training) further determines which parts of that landscape are actually explored. These two design elements are intrinsically coupled: together, they dictate both the effective search space and the set of solutions that the agent can realistically discover. Thus, these coupled design choices can make multi-objective RL significantly more brittle than the single-objective case, as small changes in scalarization or weighting can dramatically alter the reward landscape experienced by the agent [23].

Several recent works have begun to explore multi-objective reinforcement learning for de novo molecular design. MoleGuLAR [24] extends REINVENT-style fine-tuning to multi-property optimization, showing that simple weighted-sum rewards can be dominated by a subset of objectives and proposing cyclic reward schedules as a heuristic mitigation. Beyond drug discovery, Wei et al. [25] introduce a multiple-objective RL framework for inverse design problems and demonstrate that curriculum learning can accommodate a large number of objectives. More recently, Pareto-based approaches such as clustered Pareto reinforcement learning (CPRL) [26] replace scalarization altogether and directly approximate the Pareto front in property space. Furthermore, some implementations also rely on additional regularization terms. Examples include entropy bonuses to maintain diversity in the sampled molecules [17, 27], or KL-based constraints to limit overly aggressive policy updates [28], thereby tempering the impact of potentially ill-conditioned scalarized rewards. However, the majority of these methods employ limited scalarization schemes comparison (e.g. fixed weighted sums or desirability functions) or treat weight assignment heuristically, and a systematic analysis of how scalarization shape, weight schedules and regularization (e.g. KL penalties to the Prior) jointly affect exploration and Pareto coverage is still lacking.

### Our work

We explicitly focus on this joint design problem at the interface of multi-objective reward construction: how the different elements within it shape the effective reward landscape and guide the exploration behavior of the agent.

Our contributions are threefold. First, we evaluate reward scalarization methods for multi-objective molecular generation, comparing the commonly used weighted sum with product and Chebyshev scalarization across two- and three-objective target spaces. Second, we analyze weight scheduling strategies and demonstrate how dynamic weighting schemes affect RL fine-tuning behavior. Third, we derive practical recommendations for multi-objective RL pipelines in drug discovery, including the choice of scalarization function and the use of dynamic weights for a given optimization setting.

To guide the reader, the remainder of this paper is organized as follows. In section Methods, we describe the molecular generation framework, the property objectives and scoring functions used, the definitions of our reward scalarization functions, the weight assignment schemes and regularization terms implemented, as well as the experimental design and evaluation metrics. Section Results and discussion presents the outcomes of our experiments: first a baseline scenario, then sub-sections analyzing the effect of scalarization function and weight strategy. Finally, section Conclusion summarize key findings and suggest best practices and future research directions in multi-objective molecular RL.

## Methods

### Initial generative model

A molecular sequence $X = (x_0, \dots , x_T)$ can be represented either as a SMILES string [29] or as a sequence of graph-building actions [13]. In an autoregressive formulation, the probability of a sequence can be factorized into the product of conditional probabilities of each token $x_i$ given all previously generated tokens $x_{<i} = (x_0,\dots ,x_{i-1})$, under a Markovian assumption:

1 $$\begin{aligned} p(X) = \prod _{i=0}^{T} p(x_i \mid x_{<i}). \end{aligned}$$

The goal of an autoregressive generative model is to parametrize this conditional probability distribution and approximate *p*(*X*) with $p_\theta (X)$. The model parameters θ are learned by maximizing the likelihood of molecules in a training set, which in practice corresponds to minimizing the token-level cross-entropy loss under teacher forcing. For a more detailed discussion of this training procedure, we refer the reader to other works [30–32].

2 $$\begin{aligned} p(X) \approx p_\theta (X) = \prod _{i=0}^{T} p_\theta (x_i \mid x_{<i}). \end{aligned}$$

In this work, we adopt a string-based representation and train an autoregressive model over SMILES. Molecules are tokenized into a vocabulary of 43 tokens [33] (including special start- and end-of-sequence markers), and sequences are padded to a maximum length of 120 tokens. The model is implemented as an 3-layer RNN LSTM with hidden size 512 and is trained on the ChEMBL33 dataset [34] using maximum-likelihood estimation for 8 epochs, with batch size 512 and learning rate 1 $\cdot 10^{-3}$. Training and validation curves, are reported in the Supporting Information.

The trained model defines the *Prior* distribution $p^{\text {Pr}}(X) = \prod _{i=0}^{T} p_\theta (x_i \mid x_{<i})$, which serves as the starting point for the subsequent RL fine-tuning procedures described below.

### Reinforcement learning approaches for fine-tuning

Autoregressive molecular generation can be naturally viewed as a sequential decision-making problem. At each time step, the model observes a partial sequence of tokens $s_i$ representing a yet-incomplete molecule and must decide which token to append next. Under maximum-likelihood training of the Prior, this decision rule is learned so as to reproduce the empirical distribution of molecules in the training set. RL fine-tuning instead replaces this likelihood-based objective with a reward-based one that directly reflects the desired design goals.

In this setting, we frame the process as an interaction between an *Agent* (initialized with the parameters θ of the Prior) and an *Environment*. At each step, the Agent observes the current state $s_i$ (the partially built sequence) and selects an action $a_i$ (the next token) according to its policy $\pi _\theta =p_\theta ^{\textrm{Ag}}(a_i|s_i)$. The Environment implements the transition dynamics by appending this token to create the next state as $s_{i+1} = s_i \oplus a_i$. When a complete molecule *X* (a terminal state) is generated, the Environment evaluates it and issues a terminal *reward* *R*(*X*). This reward serves as a feedback signal: it is used to update the agent parameters θ so as to increase the probability of generating high-reward molecules in the future. Formally, this sequential interaction can be cast as a finite-horizon Markov Decision Process (MDP) [15, 24], where states are partial sequences, actions are tokens from the vocabulary, transitions correspond to appending tokens, and rewards are assigned only at the end of an episode. Each episode corresponds to a trajectory $\tau = (s_0, a_0, s_1, a_1, \dots , s_T, a_T)$, which is in one-to-one correspondence with a generated molecule $X = (x_0,\dots ,x_T)$. Intermediate steps receive zero reward, and a scalar terminal reward *R*(*X*) is assigned once a complete molecule has been generated.

The objective of RL fine-tuning is to maximize the expected terminal reward. In practice, we optimize this objective by minimizing its negative. Using the score-function estimator, classical policy gradient methods such as REINFORCE [17] minimize the following Monte-Carlo estimate of the negative expected reward:

3 $$\begin{aligned} L_{\textrm{REINFORCE}}(\theta )&= -\mathbb {E}_{X\sim p_\theta ^{\textrm{Ag}}}\!\left[ (R(X)-b)\,\log p_\theta ^{\textrm{Ag}}(X)\right] \end{aligned}$$

4 $$\approx \; -\frac{1}{N}\sum _{i=1}^{N} (R(X_i)-b)\,\log p_\theta ^{\textrm{Ag}}(X_i), \qquad X_i \sim p_\theta ^{\textrm{Ag}},$$

where *b* is a baseline (e.g., the batch mean reward) used to reduce gradient variance, and *R*(*X*) is constructed from the property scores of *X* (see section Scalarization approaches for multi-objective rewards). However, direct reward maximization can drift substantially from the Prior distribution [35]. For our work, we use the REINVENT formulation [36], which minimizes a squared-error objective that regresses the agent log-likelihood toward an augmented target:

5 $$\begin{aligned} L_{\textrm{REINVENT}}(\theta )&= \mathbb {E}_{X\sim p_\theta ^{\textrm{Ag}}} \Bigl [ \bigl (\log p^{\textrm{Pr}}(X) + \sigma R(X)\nonumber \\&\quad - \log p_\theta ^{\textrm{Ag}}(X)\bigr )^2 \Bigr ]\end{aligned}$$

6 $$\approx \frac{1}{N}\sum _{i=1}^N \bigl (\log p^{\textrm{Pr}}(X_i) + \sigma R(X_i) - \log p_\theta ^{\textrm{Ag}}(X_i)\bigr )^2$$

Here, $p^{\textrm{Pr}}(X)$ denotes the Prior distribution over molecules defined previously and σ controls the influence of the reward. This objective can be interpreted as encouraging the agent to match the target log-probability defined by the prior and the scaled reward. In this way, REINVENT interpolates between pure maximum-likelihood training on the Prior and pure reward maximization, while implicitly regularizing the fine-tuned policy to remain close to the Prior.

The reinforcement learning fine-tuning process utilized the Adam optimizer [37] with an learning rate set to $5\times 10^{-5}$. Training was conducted using a batch size of 64, and the maximum length for any generated molecule was restricted to 120 tokens. To ensure training stability, a gradient clip norm of 1.0 was applied. Each training run involved 3000 epochs. To establish statistical significance, each unique experimental configuration was run 5 independent times (with different seeds). In each training epoch, a batch of molecules is generated. For every molecule in the batch, the target property values are computed using RDKit 2025.03.4 [38]. These values are then mapped to a range between 0 and 1 using a desirability function. The arithmetic mean of these mapped values is calculated and subsequently aggregated (scalarized) to yield a single reward signal for the current training epoch.

### Scalarization approaches for multi-objective rewards

Given the RL formulation in section Reinforcement learning approaches for fine-tuning, the missing piece of information is the definition of the scalar reward *R*(*X*) used to evaluate each generated molecule. In realistic design settings, each molecule *X* is assessed by a set of property predictors $\textbf{f}(X) = \bigl (f_1(X), \dots , f_K(X)\bigr )$, where each $f_k(X)$ quantifies a distinct design objective (e.g. predicted target activity, physicochemical property, etc.). To make these heterogeneous quantities comparable and to reflect domain knowledge about acceptable ranges, we first transform the raw property values into dimensionless desirability scores. For each objective *k*, a desirability function $d_k$ maps the raw property value $f_k$ into a desirability score $D_k$:

7 $$\begin{aligned} d_k : \mathbb {R} \rightarrow [0,1], \qquad D_k(X) = d_k\bigl (f_k(X)\bigr ), \end{aligned}$$

where $D_k(X) \approx 0$ indicates an unacceptable value of $f_k(X)$ and $D_k(X) \approx 1$ indicates that the objective is well satisfied. In practice, the functions $d_k$ are chosen from a small set of parametric families (e.g. sigmoidal, Gaussian-like, or piecewise-linear functions), with bounds and slopes selected to reflect project-specific thresholds and tolerances. The resulting vector

8 $$\begin{aligned} \textbf{D}(X) = \bigl (D_1(X), \dots , D_K(X)\bigr ) \in [0,1]^K \end{aligned}$$

provides a normalized representation of how well *X* satisfies each design criterion. We then combine these per-objective desirability scores into a scalar score via a scalarization function

9 $$\begin{aligned} S : [0,1]^K \times \mathbb {R}_+^K \rightarrow \mathbb {R}, \qquad S= f\bigl (\textbf{D}(X), \textbf{w}\bigr ), \end{aligned}$$

where $\textbf{w} = (w_1,\dots ,w_K)$ is a non-negative weight vector encoding the relative importance of each objective. In this work, we focus on the following families:

- Weighted-sum (arithmetic mean) scalarization.
  10 $$\begin{aligned} S_{\textrm{sum}}\bigl (\textbf{D}(X), \textbf{w}\bigr ) = \sum _{k=1}^K w_k D_k(X), \end{aligned}$$
  with $\sum _{k=1}^K w_k = 1$. This corresponds to a linear scalarization of the desirability scores and emphasizes convex portions of the Pareto front. A well-known limitation is that non-convex regions of the Pareto front cannot be recovered by any choice of fixed weights.
- Weighted-product (geometric mean) scalarization.
  11 $$\begin{aligned} S_{\textrm{prod}}\bigl (\textbf{D}(X), \textbf{w}\bigr ) = \prod _{k=1}^K D_k(X)^{w_k}. \end{aligned}$$
  Unlike the weighted sum, this formulation strongly penalizes molecules that perform poorly on any single objective: a low value in one $D_k(X)$ reduces the overall product, even if other objectives are high. As a result, it promotes more balanced solutions and mitigates the risk that one high-performing objective overcompensates for a low-performing one.
- Chebyshev (Tchebycheff) scalarization. Let $\textbf{z}^* = (z_1^*, \dots , z_K^*)$ denote an ideal (utopia) point, where each $z_k^*$ represents a target desirability for objective *k*. Under a desirability function, this is set to 1. The Chebyshev scalarization is defined as
  12 $$\begin{aligned} S_{\textrm{Ch}}\bigl (\textbf{D}(X), \textbf{w}\bigr ) = \max _{1 \le k \le K} \, w_k \bigl |D_k(X) - z_k^* \bigr |. \end{aligned}$$
  This corresponds to the weighted $L_\infty$ distance to the utopia point. In the RL setting, this changed to $1 - S_{\textrm{Ch}}$ in order to reward high scoring molecules. It aims to improve the worst-performing objective first, and is able to recover solutions across the entire Pareto front, including non-convex regions, by appropriately choosing the weight vector $\textbf{w}$.

To discourage the agent from repeatedly generating very similar molecules, we modulate this scalar score with a diversity-based memory kernel [39]. Let $\mathcal {M}$ denote a memory buffer of previously generated molecules, and let

13 $$\begin{aligned} s_{\max }(X) = \max _{Y \in \mathcal {M}} \textrm{Tanimoto}(X, Y) \end{aligned}$$

be the maximum Tanimoto similarity (TS)[40] between a batch of generated molecules *X* and each Y molecules in memory $\mathcal {M}$. Using a similarity-dependent factor

14 $$\begin{aligned} \alpha (X; \mathcal {M}) = {\left\{ \begin{array}{ll} 1, & s_{\max }(X) \le \tau _{\textrm{sim}},\\ 0, & s_{\max }(X) > \tau _{\textrm{sim}}, \end{array}\right. } \end{aligned}$$

molecules that are too similar to previously generated ones (i.e. above the threshold $\tau _{\textrm{sim}}$) receive no reward.

The final scalar reward used by the RL agent is then defined as

15 $$\begin{aligned} R(X) = \alpha (X; \mathcal {M}) \, S\bigl (\textbf{D}(X), \textbf{w}\bigr ). \end{aligned}$$

Thus, $S(\textbf{D}(X), \textbf{w})$ encodes how well *X* satisfies the multi-objective design criteria, while the memory kernel $\alpha (X; \mathcal {M})$ plays the role of a binary gate, controlling the reward of molecules that are overly similar to those already found.

### Weight schemes for reward scalarization

The weight vector $\textbf{w} = (w_1, \dots , w_K)$ plays a central role in the scalarization function $S(\textbf{D}(X), \textbf{w})$, as it encodes the relative importance of the *K* objectives. In this work, we compare several strategies for assigning and updating $\textbf{w}$ during RL fine-tuning:

- Static weight assignment. A fixed weight vector $\textbf{w}$ is chosen before training and kept constant throughout fine-tuning. This is the standard setting in many MPO frameworks, where larger weights are assigned to objectives considered more important for the specific project. Static weights are simple to interpret but may bias the search towards a narrow region of the Pareto front.
- Cyclic weight assignment. In cyclic weighting, we optimize one objective at a time by using a sequence of one-hot weight vectors. For objective *k*, we set $w_k = 1$ and $w_{j \ne k} = 0$ for a predefined number of training epochs, then move to the next objective. Over a full cycle, each objective is given a dedicated optimization phase. This scheme aims to prevent any single objective from being permanently overshadowed by others.
- Random weight assignment. To encourage the discovery of diverse trade-offs, we also consider random weights sampled from a Dirichlet distribution, at training iteration (or epoch) *t*. The concentration parameters $\boldsymbol{\alpha }$ control how peaked or uniform the sampled weights are.
- Dynamic weight assignment based on performance. Finally, we introduce dynamically adapted weights that react to the observed performance of the agent on each objective. Let $\bar{D}_k^{(t)}$ denote the mini-batch mean desirability for objective *k* at iteration *t*. During training, we maintain an exponential moving average (EMA) $m_k^{(t)}$ for each objective,
  16 $$\begin{aligned} m_k^{(t)} = \lambda \, \bar{D}_k^{(t)} + (1-\lambda )\, m_k^{(t-1)}, \end{aligned}$$
  with smoothing factor $\lambda \in (0,1)$. Based on these EMAs, we consider three update rules for the weights:
  - Performance-based EMA (Regular EMA). Objectives whose EMA lags behind a utopia point value $z^*_k$ are up-weighted, while those already close to their target are down-weighted:
    17 $$\begin{aligned} w_k^{(t)} = w_k^{(t-1)} + \beta _{\textrm{EMA}}\,\bigl (z^*_k - m_k^{(t)}\bigr ), \end{aligned}$$
    where $\beta _{\textrm{EMA}} > 0$ controls the update magnitude. This scheme directs more attention to underperforming objectives.
  - Improvement-rate EMA. Here, the weight updates depend on the change in EMA between iterations:
    18 $$\begin{aligned} w_k^{(t)} = w_k^{(t-1)} - \beta _{\textrm{EMA}}\,\bigl (m_k^{(t)} - m_k^{(t-1)}\bigr ). \end{aligned}$$
    Objectives whose performance is improving rapidly (large positive $m_k^{(t)} - m_k^{(t-1)}$) have their weights reduced, whereas objectives that stagnate or deteriorate are up-weighted, encouraging the agent to focus on objectives that are not yet progressing.
  - Variance-based EMA. Finally, we investigate using the empirical variance of the EMA to modulate the weights. Let $\sigma _k^2(t)$ denote the variance of $m_k^{(\cdot )}$ over a sliding window of recent iterations. We update
    19 $$\begin{aligned} w_k^{(t)} = w_k^{(t-1)} + \beta _{\textrm{EMA}}\, \sigma _k^2(t)\,, \end{aligned}$$
    so that objectives with more volatile performance receive higher weights. This encourages the agent to stabilize objectives that fluctuate strongly.
  After each update, we project $\textbf{w}^{(t)}$ back onto the probability simplex (i.e. enforce $w_k^{(t)} \ge 0$ and $\sum _k w_k^{(t)} = 1$), ensuring that the weights remain interpretable as relative importance factors.

The cyclic weight assignment scheme was configured with a cycle length of 50 epochs. The random weight assignment scheme sampled new weights every epoch based on a Dirichlet distribution parametrized by α=1. For the Exponential Moving Average (EMA) schemes, updates to the weights occurred every 5 epochs. The smoothing parameter α was set to 0.8. The β parameters used across the different EMA variants were: 10 for the performance and improvement rate variants, and 100 for the variance method.

In summary, we consider both static weight vectors $\textbf{w}$ (fixed throughout training) and time-varying schedules $\{\textbf{w}^{(t)}\}$ that progressively re-balance the importance of different objectives over the course of fine-tuning. This enables us to probe how the choice of scalarization function and weight assignment scheme jointly influence the structure of the reward landscape and the regions of the Pareto front that are effectively explored by the agent.

### Entropy regularization

Scalarization functions and weight choices can induce sharply peaked or rugged reward landscapes, which can in turn drive the agent to collapse onto a narrow subset of chemical space. To mitigate this, we explore the addition of an entropy bonus to the training loss at the sequence level to encourage exploration during sequence generation.

For a discrete action space $\mathcal {A}$, the (per-step) policy entropy is

20 $$\begin{aligned} H(\pi _\theta (\cdot \mid s)) = -\sum _{a\in \mathcal {A}}\pi _\theta (a\mid s)\log \pi _\theta (a\mid s). \end{aligned}$$

Given sampled sequences $X_i\sim p_\theta ^{\textrm{Ag}}$ with corresponding state sequences $\{s_{i,t}\}_t$ and padding mask $m_{i,t}$, we define the entropy-regularized loss as

21 $$\begin{aligned} & L_{\textrm{reg}}(\theta ) = L_{\textrm{RL}}(\theta ) - \beta \, \mathbb {E}_{X\sim p_\theta ^{\textrm{Ag}}} \nonumber \\ & \quad \Bigl [ \sum _t m_t \, H\bigl (\pi _\theta (\cdot \mid s_t)\bigr ) \Bigr ], \qquad \beta \ge 0, \end{aligned}$$

Larger values of β encourage more stochastic policies and help maintain diversity in the generated molecules. Thus, minimizing $L_{\textrm{reg}}$ encourages more stochastic policies.

### Evaluation metrics

By systematically varying the scalarization function *S*, the weight vector $\textbf{w}$, and the regularization scheme, we can characterize how these design choices jointly shape the effective reward landscape and the exploration behavior of the agent.

To compare these different schemes, we use the hypervolume (HV) indicator as our primary measure of multi-objective performance [41]. Given a set of non-dominated solutions in objective space and a fixed reference point, the HV corresponds to the volume of the region that is dominated by the solution set and bounded by the reference point. In our setting, we compute HV over the normalized objective (or desirability) values with a common reference point shared across all methods. Higher HV values indicate that the obtained solutions are both closer to the (unknown) true Pareto front and cover a larger portion of it. By tracking HV as a function of training epochs, we can directly quantify how quickly different scalarization and weighting schemes guide the agent toward high-quality trade-offs in objective space.

Our framework utilizes the implementation of the Nowak et al. [42] algorithm for HV computation, as provided by the *pygmo* 2.19.7 Python package. In our setting, we compute HV over the normalized objective values with a common reference (*Nadir point*) shared across all methods. Given the objective of maximization for all criteria, the reference point is chosen such that its values are less than or equal to the normalized objective values of all considered non-dominated solutions. As a diversity measure we are utilizing the pairwise Tanimoto similarity, implemented in RDKit 2025.03.4 [38].

#### Diversity assessment metrics

To assess the structural variety and distribution of the generated chemical space, we use two complementary metrics: Tanimoto similarity (TS) [40] to characterize the distribution of pairwise molecular similarity, and normalized Shannon entropy (NE) [43, 44] to quantify scaffold diversity.

Concretely, molecular similarity is computed via the Tanimoto coefficient *s*(*x*, *y*), which measures the overlap between the binary fingerprint (bit-vector) representations of two molecules *x* and *y*:

22 $$\begin{aligned} s(x,y)=\frac{c}{a+b-c}, \end{aligned}$$

where *a* and *b* denote the numbers of set bits in the fingerprints of *x* and *y*, respectively, and *c* is the number of bits set in both. We refer to TS as the empirical distribution of these pairwise coefficients over a set of (sampled) molecule pairs, $\textrm{TS}=\{\,s(x_i,x_j)\mid (i,j)\in \mathcal {P}\,\},$ where $\mathcal {P}$ denotes the set of considered molecule pairs.

For evaluation, 5000 molecules were sampled every 150 epochs from epoch 150 to 3000 for each target-scalarization configuration. This procedure was repeated across five independent runs, yielding 25,000 molecules per checkpoint; the TS distribution was then estimated from the corresponding set of pairwise *s*(*x*, *y*) values. For all experiments, TS is computed using Morgan fingerprints with radius 2 and 2048-bit vectors.

While TS summarizes overall molecular similarity across generated compounds, NE measures the uniformity of the scaffold distribution, allowing us to determine whether the model collapses onto a small number of recurring frameworks or instead explores a broader range of scaffold classes.

Let $\{c_i\}_{i=1}^{n}$ be the counts of the *n* unique scaffolds observed in a generated batch and define the empirical scaffold distribution $p_i = c_i/\sum _{j=1}^{n} c_j$. The Shannon entropy of the scaffold distribution is

23 $$\begin{aligned} H(p) = -\sum _{i=1}^{n} p_i \log p_i , \end{aligned}$$

and we report the normalized Shannon entropy

24 $$\begin{aligned} NE = \frac{H(p)}{\log n}, \end{aligned}$$

where logn is the maximum attainable entropy (achieved when $p_i=1/n$). By convention, we set NE=0 when n=1.

## Results and discussion

In this section, we analyze the role of scalarization methods and weight assignment schemes, tracking reward, hypervolume (HV), and policy entropy throughout reinforcement learning fine-tuning.

We first consider two-objective problems, where trade-offs can be interpreted more directly, and then extend the analysis to three-objective settings. The implications of these observations for higher-dimensional objective spaces are discussed separately in section Implications for higher-dimensional objective spaces.

### Two-objective optimization

We begin with optimization in the log*P*-synthetic accessibility (SA) property space. This two-objective setting provides a controlled test case for comparing scalarization behavior, since the resulting reward landscapes and generated molecular distributions can be inspected directly.

#### Impact of scalarization

To assess how scalarization affects policy updates, we defined three target regions using Gaussian desirability functions in the log*P*-SA space. For each property, the desirability approaches 1 near the target value and decreases with distance from it. The resulting property-wise desirabilities were then combined into a scalar reward using one of three scalarization functions: (i) arithmetic mean (sum), (ii) geometric mean (product), and (iii) Chebyshev-type scalarization. In this first comparison, both objectives were assigned equal static weights.

**Fig. 1:**
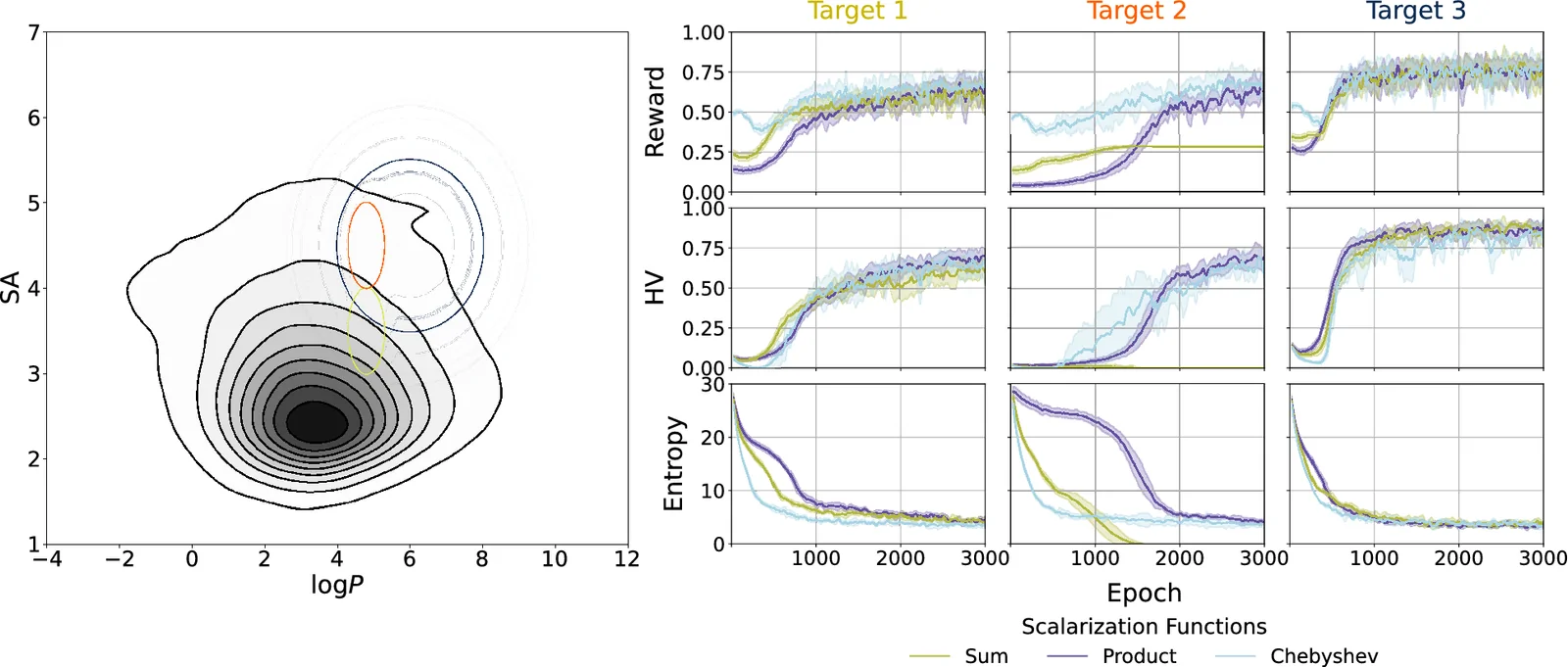
Training progression statistics for the three target regions. The left panel illustrates the KDE of the training data of the prior model against the targets (indicated by the yellow, orange and dark blue areas). Darker shades of the KDE indicate a higher probability density. The right panel details the evolution of average reward (top row), hypervolume (middle row) and token-level entropy (bottom row) over the course of fine-tuning

Figure 1 summarizes the kernel density estimates (KDEs) of the prior distribution, the three target regions, and the corresponding learning dynamics in terms of reward, hypervolume (HV), and entropy. Quantitative alignment metrics between the prior and each target (Mahalanobis distance, prior density at the target, and average Wasserstein distance) are reported in Table S1.

***Target-wise training dynamics***

Target 1 defines a narrow desirability region close to the prior distribution, assessed by highest prior density and shortest Mahalanobis distance (see Table S1. In this setting, all three scalarization methods successfully optimized the policy, with performance differences according to scalarization (Fig. 1, yellow).

All three methods achieved comparably similar rewards, only changing the number of epochs needed to reach said values. Chebyshev and product reached rapidly, while product caught up at later epochs. Albeit the HV values showed minor differences in the first 1000 epochs, at later stages both product and Chebyshev attained higher HV than sum, indicating a more comprehensive coverage of the multi-objective trade-off surface. The cumulative token-level policy entropy revealed distinct exploration–exploitation profiles. Chebyshev showed the steepest entropy decline, rapidly concentrating the policy in a narrow high-reward region. Sum and product exhibited slower entropy decay (around epochs 500 and 700, respectively), converging to similar entropy levels at later stages. Overall, for a narrow yet close target distribution, all scalarizations are viable, but nonlinear approaches such as Chebyshev and product tend to yield better performance.

Target 2 retains the same narrow distribution of Target 1 but is located farther from the prior. This configuration is thus more demanding: the policy must shift to a tightly constrained region poorly supported by the prior. In this regime, the behavior of the scalarization methods diverged sharply. A pronounced failure mode emerged for the sum scalarization: around epoch 1000, the reward stagnated, and the HV and sequence entropy collapsed (Fig. 1, orange), indicating policy collapse into a suboptimal local optimum. In contrast, both product and Chebyshev scalarization continued to optimize successfully. Their HV trajectories likewise rose to values comparable to Target 1, albeit at later epochs, indicating that said policies captured a similarly large high-quality region in molecular property space under the selected reference point. However, these scalarization methods reached this goal via different exploration–exploitation trade-offs: Chebyshev rapidly decreased token-level entropy, whereas product showed a substantial delay. This suggests that geometric mean (product) preserves exploration for longer before concentrating on the high-reward region. The comparison between Targets 1 and 2 shows how optimization difficulty increases as the target moves away from the prior distribution, and exposes the weakness of sum scalarization compared with product or Chebyshev schemes.

Target 3 is located at a larger distance from the prior with respect to Target 2, albeit with a broader desirability region. This relaxes the constraints on optimization, providing multiple high-reward directions in property space. In sharp contrast to Target 2, all three scalarization methods behaved similarly for Target 3. Reward trajectories converged to a comparable level for sum, product, and Chebyshev, surpassing the results for the other targets. The HV curves closely overlapped over the entire training horizon, with a slight improvement for sum and product relative to Chebyshev (Fig. 1, dark blue). Entropy decayed rapidly during the first 500 epochs for all scalarizations and then plateaued at comparable values across methods. Thus, when the target region is broad, the distance from the prior has a reduced effect on optimization difficulty, and the choice of scalarization function does not substantially alter training dynamics or final performance.

***Reward landscapes and optimization trajectories***

**Fig. 2:**
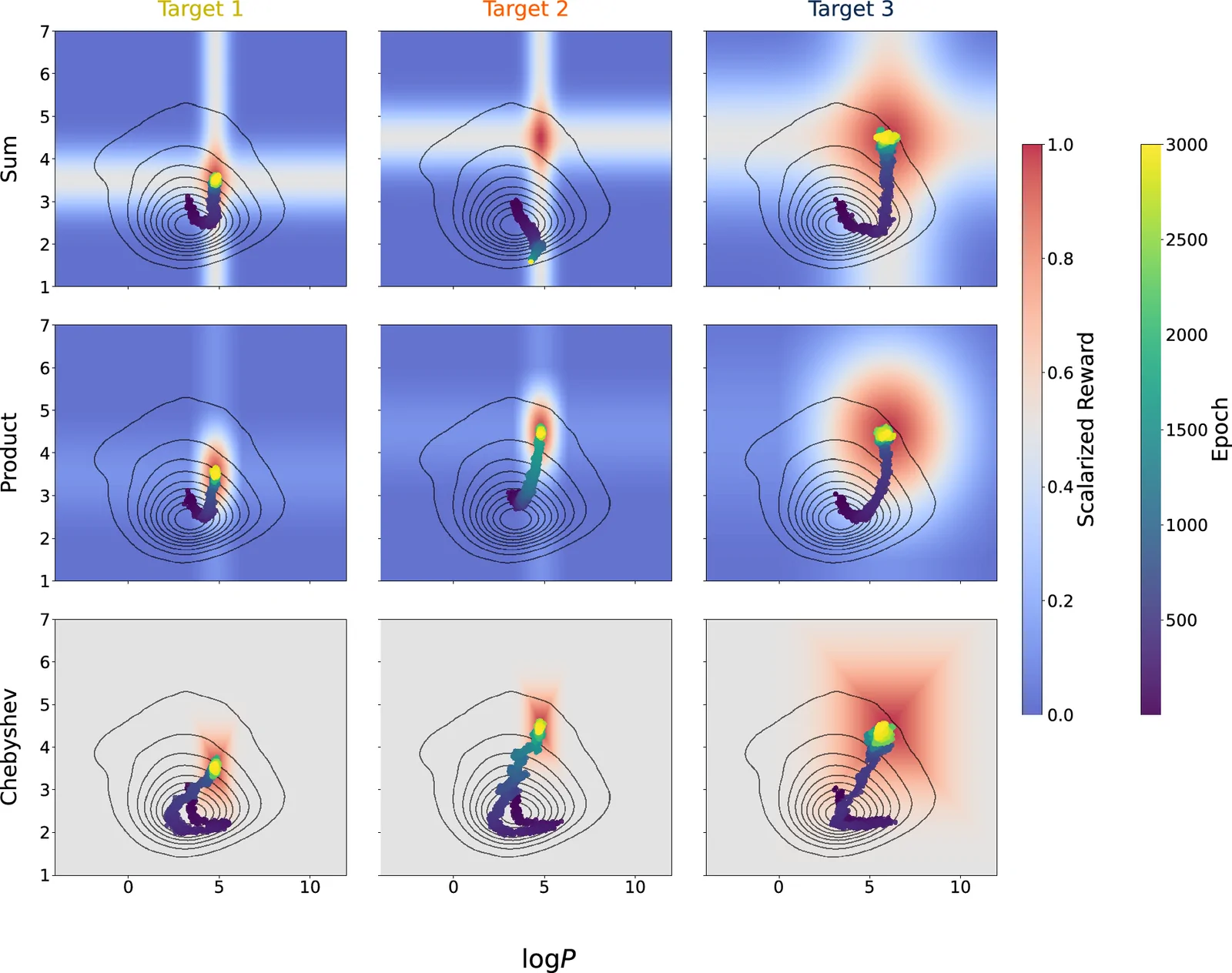
Scalarized reward landscapes and optimization trajectories in property space. Each panel shows the scalarized reward surface in (log*P*, SA) space for a given target and scalarization method (background colormap), overlaid with the trajectory of batch-wise mean properties of molecules generated during RL fine-tuning (markers across epochs, ranging from dark blue to yellow). Columns correspond to targets (Target 1–3) and rows to scalarization methods (sum, product, Chebyshev)

To gain a mechanistic understanding of the training dynamics observed across targets and scalarization methods, we analyzed the scalarized reward landscapes together with representative optimization trajectories. Figure 2 visualizes for each target (columns: Target 1, Target 2, Target 3) and scalarization method (rows: sum, product, Chebyshev), the scalarized reward surface in (log*P*, SA) space overlaid with the trajectory of sampled properties over the course of training.

Across all targets, the location of the highest-reward region is, by construction, centered around the target intersection. However, the scalarization methods differ substantially in how they shape the surrounding medium- and low-reward regions, which in turn determines which *paths* are attractive during optimization. Under sum scalarization, broad ridges of intermediate reward extend away from the target region along the coordinate axes. Because additive scalarization allows one objective to compensate for a deficiency in the other, these ridges create attractive ascent directions that can be dominated by improving a single, more accessible objective. In contrast, product scalarization strongly suppresses such compensatory ridges: because the total reward approaches zero whenever any individual desirability is low, regions that sacrifice one objective become much less rewarding. Chebyshev scalarization reshapes the landscape in a different way: the scalar reward is largely governed by the worst-performing objective, producing broad regions with similar scores when one dimension remains limiting, while sharply rewarding configurations that approach the joint optimum.

We next consider how these landscapes translate into actual optimization trajectories. Across most settings, trajectories exhibit an initial exploratory phase in which the policy moves toward the closest region of nontrivial reward under the induced landscape, followed by a slower refinement phase that attempts to approach the high-reward basin around the target intersection. This behavior differs systematically by scalarization. Under sum scalarization, trajectories often move rapidly onto an intermediate-reward ridge early in training and then continue to optimize predominantly near that ridge, reflecting the strong gradients created by compensatory reward structure. Under product scalarization, the attenuation of these ridges reduces the incentive to over-optimize a single objective; trajectories therefore tend to move more directly toward regions that satisfy both objectives simultaneously. Under Chebyshev, the reward landscape becomes mostly flat except in the target center; trajectories consequently are free to cover a broader area during training in order to find solutions to escape from the limiting dimension.

A critical failure mode becomes apparent for Target 2 under sum scalarization. In this case, the trajectory drifts along an intermediate-reward ridge at approximately constant log*P* and progressively moves away from the target region prior to collapse. This counterintuitive drift is a direct consequence of linear compensation: when the prior distribution is distant from the joint target, the steepest-ascent direction of the scalarized reward can favor maximizing a single accessible objective rather than moving toward the intersection that jointly satisfies both. The policy is thus drawn into a local optimum dominated by finding the ideal log*P*, and once the reward signal changes only minimally along the visited ridge, optimization stalls. This stall precedes the stagnation in reward and HV and the rapid entropy collapse observed in Fig. 1.

**Fig. 3:**
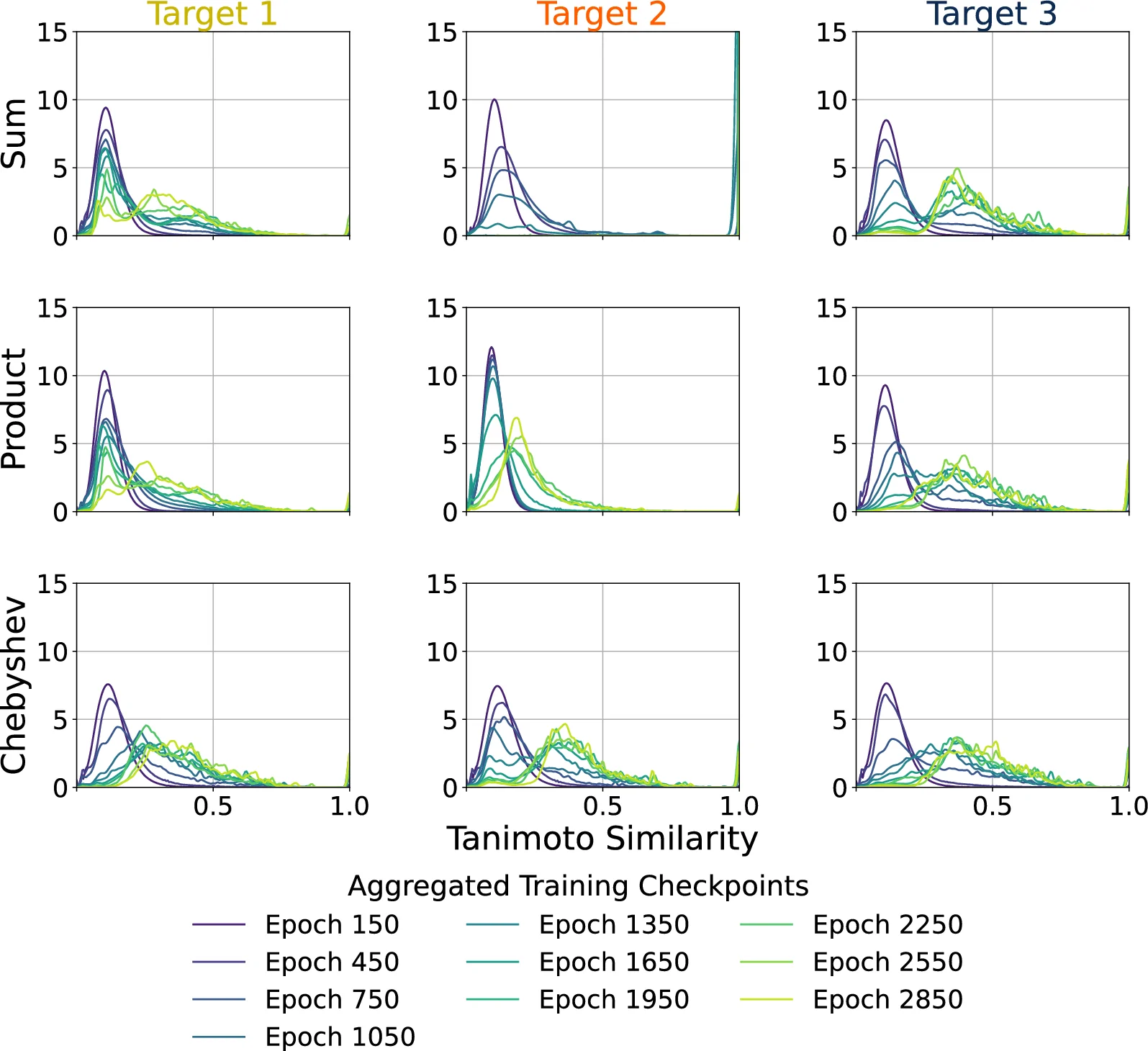
Evolution of Tanimoto similarity distributions under RL fine-tuning for an averaged run. Each panel shows the KDE of the Tanimoto similarity distributions for a target region (columns) across multiple training checkpoints (color-coded by epoch). Comparisons are shown across three scalarization methods: sum (top), product (middle), and Chebyshev (bottom)

Taken together, these landscape-and-trajectory analyses explain why scalarization choices have minimal impact for broad targets yet become decisive in constrained, low-support regimes. When the desirability region is broad, nonzero rewards are available across a larger portion of property space, and all three scalarizations produce stable trajectories with similar outcomes. As targets become narrower and the reward landscape effectively sparser, scalarization critically shapes which ascent directions are available: Chebyshev tends to drive rapid early progress by emphasizing the limiting objective, product preserves robustness by discouraging imbalance, and sum is most susceptible to ridge-following behavior (Fig. 3).

***Diversity analysis***

**Fig. 4:**
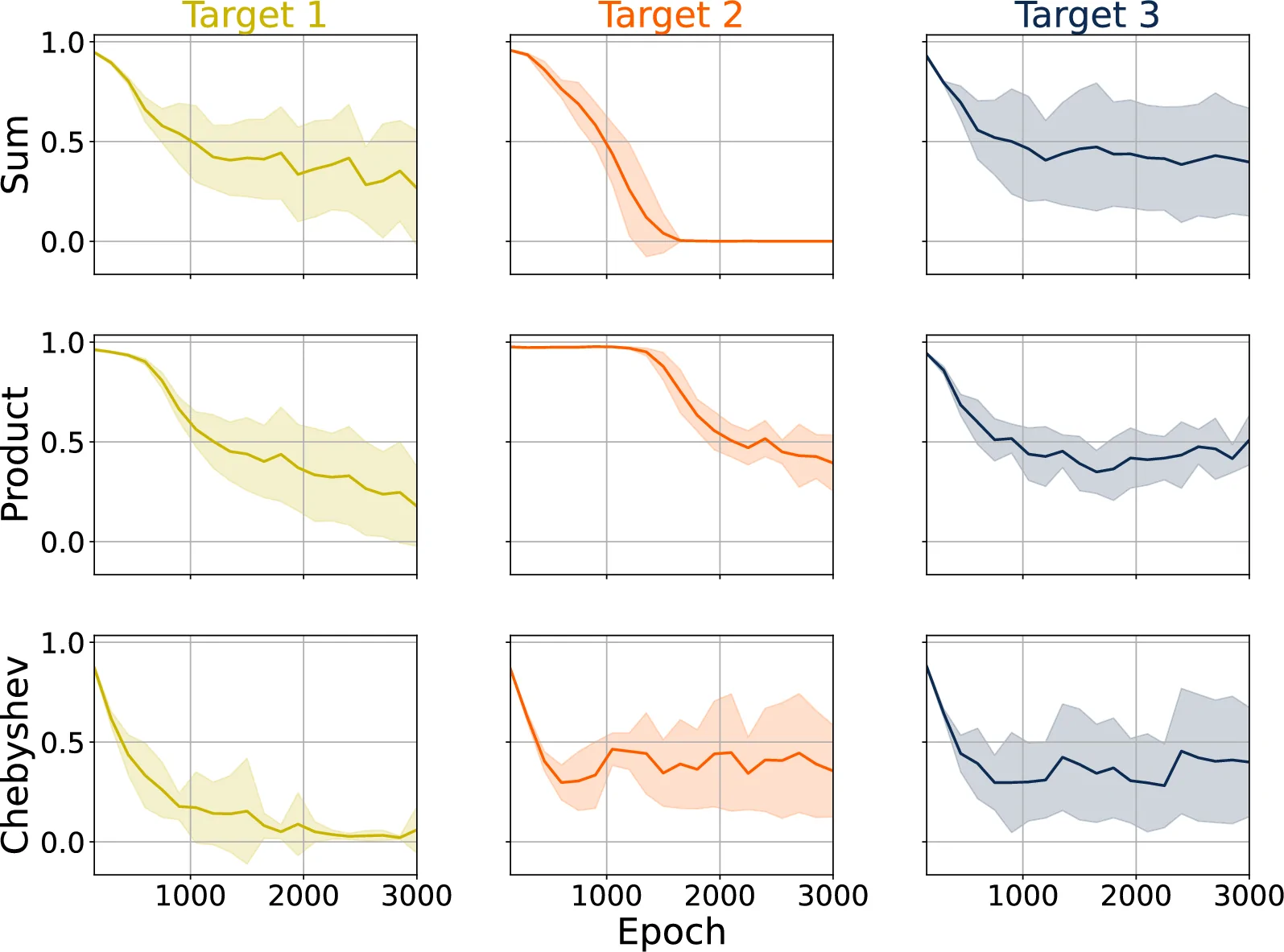
Evolution of NE of Bemis-Murcko scaffolds during RL fine-tuning. Rows correspond to scalarization methods (Sum, Product, Chebyshev) and columns to target regions. Shaded areas represent the 95% confidence interval

To understand the structural impact of scalarization during the fine-tuning process, we analyzed the evolution of the generated chemical space using two metrics: pairwise Tanimoto Similarity of Morgan fingerprints to assess molecular-level diversity (Fig. 9), and Normalized Shannon Entropy of Bemis-Murcko scaffolds to quantify scaffold diversity (Fig. 4).

First, we monitored the internal diversity of the generated libraries using pairwise Tanimoto similarity (Fig. 9). We sampled 5000 molecules at each 150 epochs for every target-scalarization function combination. At early training stages, all regimes exhibit a pronounced density peak at $\textrm{TS}\approx 0.15$, reflecting the high structural diversity induced by the prior distribution. As the policy converges toward the high-reward target regions of the chemical space, these distributions also shift toward higher similarity values, indicating that policy improvement is accompanied by a reduction in molecular diversity. However, the magnitude and shape of this shift depend strongly on the scalarization strategy and the target region.

For Target 1, which is narrow and close to the prior, both sum and product scalarizations shift gradually to higher TS values at later epochs, characterized by a heavy tail extending toward higher similarity. Although the original low-similarity mode remains visible, a second mode emerges for both methods at around 0.3, with the mode under sum scalarization slightly shifted toward higher similarity. In contrast, under Chebyshev scalarization the entire distribution transports itself towards higher similarity values, indicating a more uniform drift toward structurally similar compounds.

Target 2, which is narrow but more distant from the prior compared to Target 1, shows the most pronounced difference between scalarization methods. Under sum scalarization, a bimodal distribution emerges after epoch 1000, rapidly evolving into a sharp, high-density spike at TS close to 1.0. This represents a complete mode collapse, where the model generates nearly identical structural motifs to maximize the reward. This collapse was observed in all the runs. On the other hand, product and Chebyshev scalarizations maintain significantly higher diversity. The product method converges to a stable, localized peak at TS≈0.2, while Chebyshev produces a broader distribution centered at TS≈0.4 with a heavy tail towards larger similarity values.

For Target 3, which is broad and distant from the prior, the TS value distributions across the three scalarization methods show the least pronounced difference. Across all methods, the transition follows a consistent pattern: the initial density peak at TS≈0.15 gradually attenuates as a broader secondary distribution emerges within the 0.3 to 0.5 range. The most notable distinction occurs in the late-stage training epochs; sum scalarization yields a more localized and pronounced density peak centered at TS≈0.4. In contrast, the distributions produced by product and Chebyshev scalarization remain comparatively more diffuse.

The evolution of the Tanimoto similarity density across epochs provides a complementary view to the performance metrics observed earlier. Product scalarization consistently yields the most diverse molecular sets, characterized by smoother, broader KDEs that transition slowly from a given prior. This can be ascribed to the multiplicative constraint, which prevents the policy from over-optimizing a single objective through a narrow set of chemical structures. This suggests that this scalarization is the one that better combines chemical space exploration with improving the reward rather than rapidly exploiting a specific structural mode. Chebyshev scalarization seemed to yield a similar TS distribution across targets, probably due to its capacity to optimize sequentially its objectives.

However, pairwise similarity metrics alone do not fully distinguish whether increased similarity arises from collapse onto a small number of recurring frameworks or from local exploration within many distinct chemotypes. Therefore, we additionally quantified scaffold diversity using the normalized Shannon entropy (NE) of the generated Bemis–Murcko scaffolds [44]. For that purpose, we sampled 10,000 molecules at each 150 epochs for every target-scalarization function combination and extracted the distribution of scaffolds to calculate the NE (Fig. 4). To assess sampling convergence, we additionally report the same analysis using 100k sampled molecules in the Supporting Information (Figure S17). An NE value of 1.0 indicates a uniform distribution of over unique scaffolds, while a value of 0.0 indicates complete mode collapse to a single scaffold.

For Target 1, all scalarization methods exhibit a decline in scaffold diversity as the model adapts to the target region, but the rate of decay differs. The Chebyshev scalarization (bottom-left) shows the most rapid loss of scaffold diversity, with NE dropping sharply below 0.2 within the first 1000 epochs and remaining essentially static thereafter. In contrast, sum and product scalarizations maintain moderate scaffold diversity for longer, stabilizing between 0.25 and 0.4. This suggests that for targets close to the prior, Chebyshev scalarization aggressively prunes the scaffold space, while sum and product retain a broader variety of chemotypes.

For Target 2, narrow but distant from the prior, sum scalarization (top-middle), the scaffold entropy undergoes a catastrophic collapse, hitting NE≈0.0 shortly after epoch 1500. This confirms that the high Tanimoto similarity observed previously corresponds to the model fixating on a single structural skeleton. Conversely, both product and Chebyshev scalarizations resist this collapse. Product scalarization in particular, exhibits high NE during the first 1200 epochs and stabilizes at a significantly higher NE≈0.5 compared to the other scalarization functions. Chebyshev exhibits fluctuating behavior, settling near NE≈0.4.

For Target 3 which is broad and distant from the prior, the distinctions between methods are small, which is consistent with previous trends and with the findings for the average cumulative entropy in Fig. 1. All methods experience a reduction in scaffold diversity, stabilizing between 0.3 and 0.5. Product scalarization again demonstrates the most robust diversity preservation, maintaining a steady NE around 0.5 with tighter confidence intervals than the sum scalarization. This reinforces the observation that multiplicative scalarization encourages the exploration of multiple distinct solutions rather than exploiting a single path.

Overall, the scaffold diversity analysis highlights a distinct “hierarchy of decay." Chebyshev scalarization generally decays much faster than the other two methods, driving the policy quickly toward specific high-performing scaffolds. Consequently, it produces the lowest number of unique scaffolds across the training run. In contrast, product scalarization takes the longest to explore the chemical space, reflected in a higher normalized entropy that persists for a longer duration. This sustained exploration also results in the highest total number of unique generated scaffolds. Sum scalarization typically falls between these extremes, except in cases of mode collapse (Target 2). This suggests that while long-term convergence values may be similar for easy targets, the choice of scalarization significantly dictates the breadth of chemical space conserved during the training process. A figure of the top five generated scaffolds and the total unique scaffold counts for each method is provided in the Supporting Information (Figures S14-S16).

#### Influence of weight scheduling strategies

Following the established performance differences among the scalarization methods, we investigated how different weight scheduling strategies interact with these scalarizations. We considered three classes of schemes: (i) a *static* strategy (the baseline from the previous section), (ii) dynamic strategies independent of target performance (*random* and *cyclical* assignment), and (iii) dynamic strategies explicitly dependent on the target (*performance*-, *improvement*-, and *variance*-based schemes). These methods are described in detail in section Weight schemes for reward scalarization. All schemes were applied across the three target regions with different levels of difficulty introduced in section Impact of scalarization. The results are visualized in terms of average reward, HV, and average batch entropy over the course of training.

**Fig. 5:**
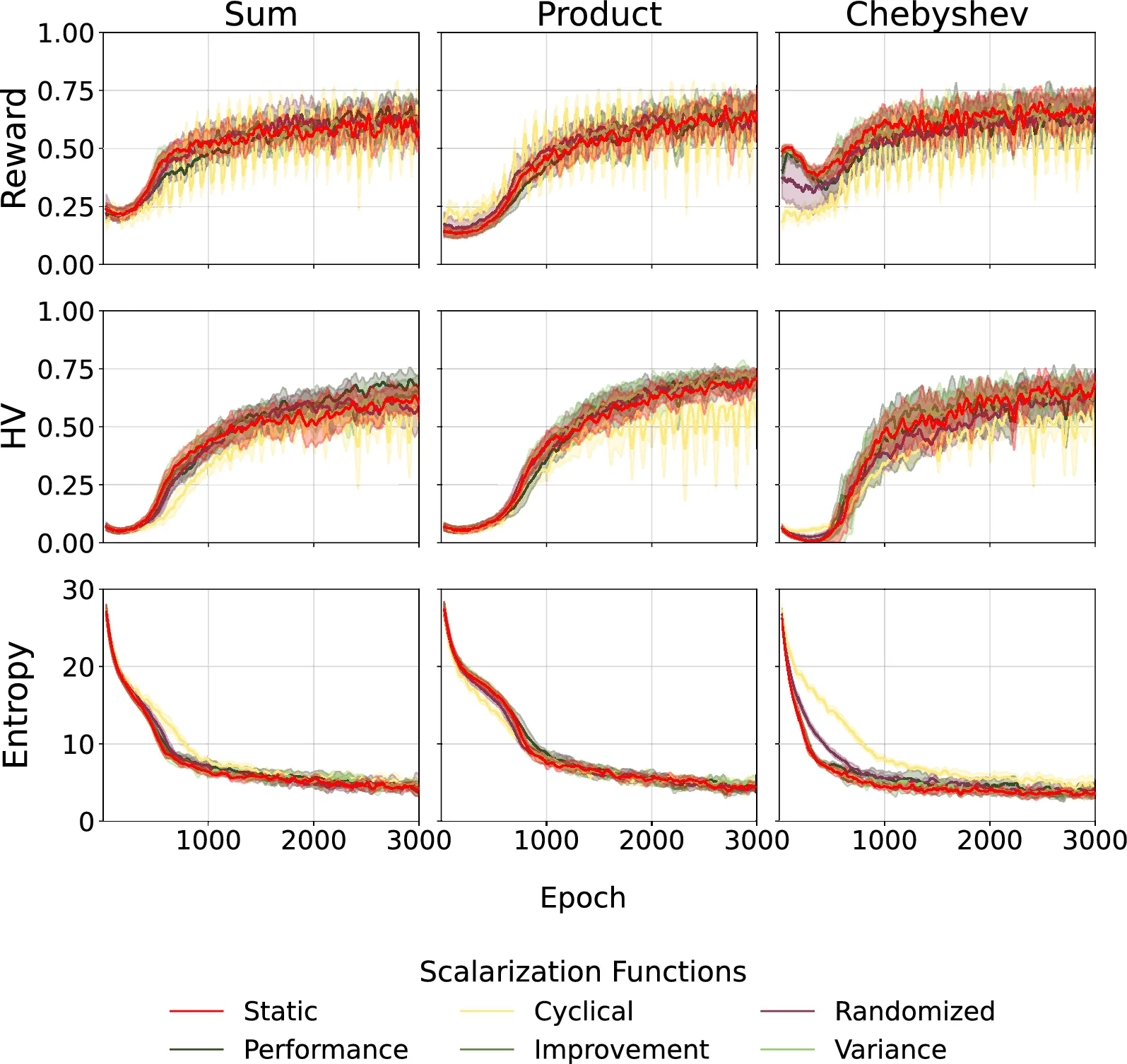
Effect of weight scheduling strategies on RL fine-tuning dynamics across scalarization methods for Target 1. Columns correspond to scalarization functions (sum, product, Chebyshev), and rows report the evolution of average reward (top), hypervolume (middle), and batch entropy (bottom) over training epochs. Within each panel, curves for all weight schedules (*static*, *random*, *cyclical*, *performance*, *improvement*, *variance*) are overlaid to facilitate direct comparison

For Target 1, the training dynamics for all scalarization methods and weight schedules are shown in Fig. 5. For sum scalarization, all weighting schemes converged to an average reward of approximately 0.6, comparable to the *static* baseline. The *cyclical* scheme occasionally reached higher reward values, but exhibited oscillatory behavior reflecting its periodic schedule. In terms of HV, *performance* slightly outperformed the others in the long run (epochs > 2000). The remaining target-dependent schemes (*improvement* and *variance*) achieved similar HV values, while *cyclical* weighting resulted in a slightly lower HV than the other methods. Entropy decay was broadly similar across schemes, with *cyclical* showing a slightly slower decay.

For product scalarization, reward trajectories started at a notably lower value and increased more slowly than under sum or Chebyshev. The HV curves showed only minor differences between weighting methods, with the exception of *cyclical*, which underperformed. This pattern was reflected in the entropy decay: all schemes exhibited relatively slow entropy reduction, with *cyclical* in the lower tier.

For Chebyshev scalarization, more pronounced differences emerged in the reward trajectories. During the first ≈500 epochs, the *static*, *improvement*, *variance*, and *performance* schemes showed a similar initial dip followed by recovery, all outperforming *random* and *cyclical* weighting. At later stages, most methods achieved comparable reward levels, with *cyclical* lagging significantly behind. HV values suggested that *random* and *cyclical* weighting performed worst, whereas the *static* and target-dependent schemes performed comparably better. This was mirrored in the entropy profiles: *random* and *cyclical* weights maintained higher entropy for longer and decayed more slowly than the other schemes.

Overall, for Target 1 we observed that target-independent dynamic approaches such as *random* and *cyclical* weighting tended to perform slightly worse than the target-dependent or *static* schemes, especially under Chebyshev scalarization. Consistent with the previous section, product scalarization generally achieved higher HV than sum or Chebyshev when combined with well-behaved weighting schemes.

**Fig. 6:**
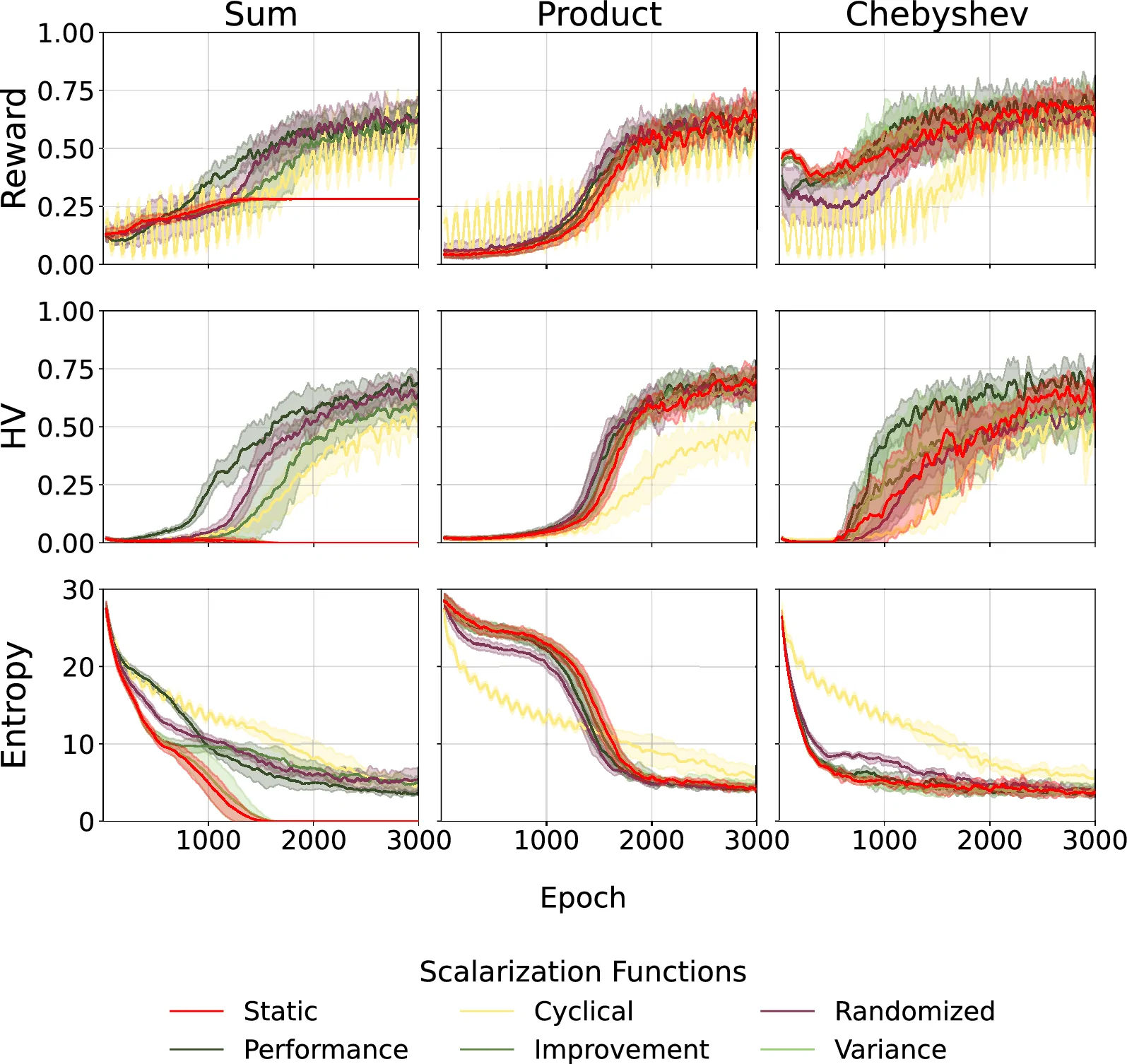
Effect of weight scheduling strategies on RL fine-tuning dynamics across scalarization methods for Target 2. Columns correspond to scalarization functions (sum, product, Chebyshev), and rows report the evolution of average reward (top), hypervolume (middle), and batch entropy (bottom) over training epochs. Within each panel, curves for all weight schedules (*static*, *random*, *cyclical*, *performance*, *improvement*, *variance*) are overlaid to facilitate direct comparison

In comparison, for Target 2, the training dynamics are illustrated in Fig. 6. For sum scalarization, a pronounced failure mode was observed for the *static* weight assignment, as reported in section Impact of scalarization. A similar collapse occurred under the *variance*-based scheme. In both cases, the failure consistently appeared around epoch 1200. In sharp contrast, the other weight assignment methods were able to prevent this collapse. The *performance* scheme achieved the highest reward up to approximately epoch 1600; subsequently, *random* weighting caught up, and both schemes outperformed the *improvement*-based and *cyclical* methods (which lagged until around epoch 2200). The HV analysis highlights the efficiency of the *performance* scheme: it reached an HV of 0.5 by epoch 1600, substantially faster than *random* (around epoch 1900), *improvement*-based (around epoch 2200), or *cyclical* weighting (around epoch 2800). Entropy decay was strongest (fastest) for *static* and *variance*, followed by *performance*, while *random* and *improvement* weights showed similar, more gradual decay.

For product scalarization, reward differences between weighting schemes were less pronounced than for sum or Chebyshev. The *performance* and *random* schemes yielded slightly higher rewards between epochs 1000–1700, which was reflected in their HV curves. In the long run, most schemes achieved comparable HV values, except for *cyclical*, which remained significantly worse. The entropy dynamics showed the characteristic behavior described in section Impact of scalarization: entropy decreased slowly for the first ≈1000 epochs for all schemes, with *cyclical* as a clear outlier. Among the remaining methods, *static* and *variance* weights maintained higher entropy for longer and decreased rapidly only in late stages.

For Chebyshev scalarization, differences between weight schedules were most pronounced. *Cyclical* weighting led to the lowest rewards, followed by *random*. The *static*, *variance*, and *improvement* schemes achieved higher rewards early in training (epochs 0–300), but were overtaken by the *performance* scheme around epoch 1000. In terms of HV, *performance* clearly dominated, followed by *variance*-based, *improvement*-based, and *static* weighting. *Random* and *cyclical* schemes yielded consistently lower HV than the target-dependent strategies. Entropy-wise, most schemes exhibited comparable decay profiles, except for *random* and *cyclical* weighting, which again showed slower decay and maintained higher entropy for longer.

For completeness, we also evaluated all weighting strategies on Target 3. In line with the results of section Impact of scalarization, the broader and less constrained target made the optimization problem relatively forgiving: none of the weighting schemes produced substantial differences in reward, HV, or entropy for any of the three scalarization methods. Detailed curves for Target 3 are provided in Figure S4. This further supports the observation that both scalarization choice and weight scheduling become critical only when the target region is narrow and weakly supported by the prior.

Overall, we observed that the *performance* scheme achieved consistently better performance than the other weight assignment strategies across scalarization methods, particularly in the more challenging Target 2 setting. The superior behavior of the *performance* weighting can be attributed to its formulation: it is the only scheme that explicitly incorporates the target value $z^{*}$ into the weight update rule, $w_k(t) \propto z_k^{*} - m_k(t)$. By scaling weights in proportion to the distance from the desired goal, it naturally intensifies the optimization pressure on objectives that are lagging behind. In the context of Chebyshev scalarization, which already focuses on the worst-case objective, this synergy is particularly effective: the weighting method aligns the optimization focus with the dimension that the scalarization penalizes most strongly, leading to the rapid HV growth observed for Target 2.

The model collapse observed with *static* and *variance*-based weights under sum scalarization (Target 2) suggests that rigid or purely stability-oriented weights are insufficient for navigating the loss landscape when objectives are strongly competing and the reward signal is initially sparse. *Variance*-based weighting, which increases weights for volatile objectives, may inadvertently amplify noise instead of signal during critical learning phases, thereby accelerating collapse rather than preventing it. In contrast, performance-aware schemes that adapt weights toward unmet objectives provide a more reliable signal for guiding the policy out of local optima induced by the scalarization.

#### Influence of entropy regularization

**Fig. 7:**
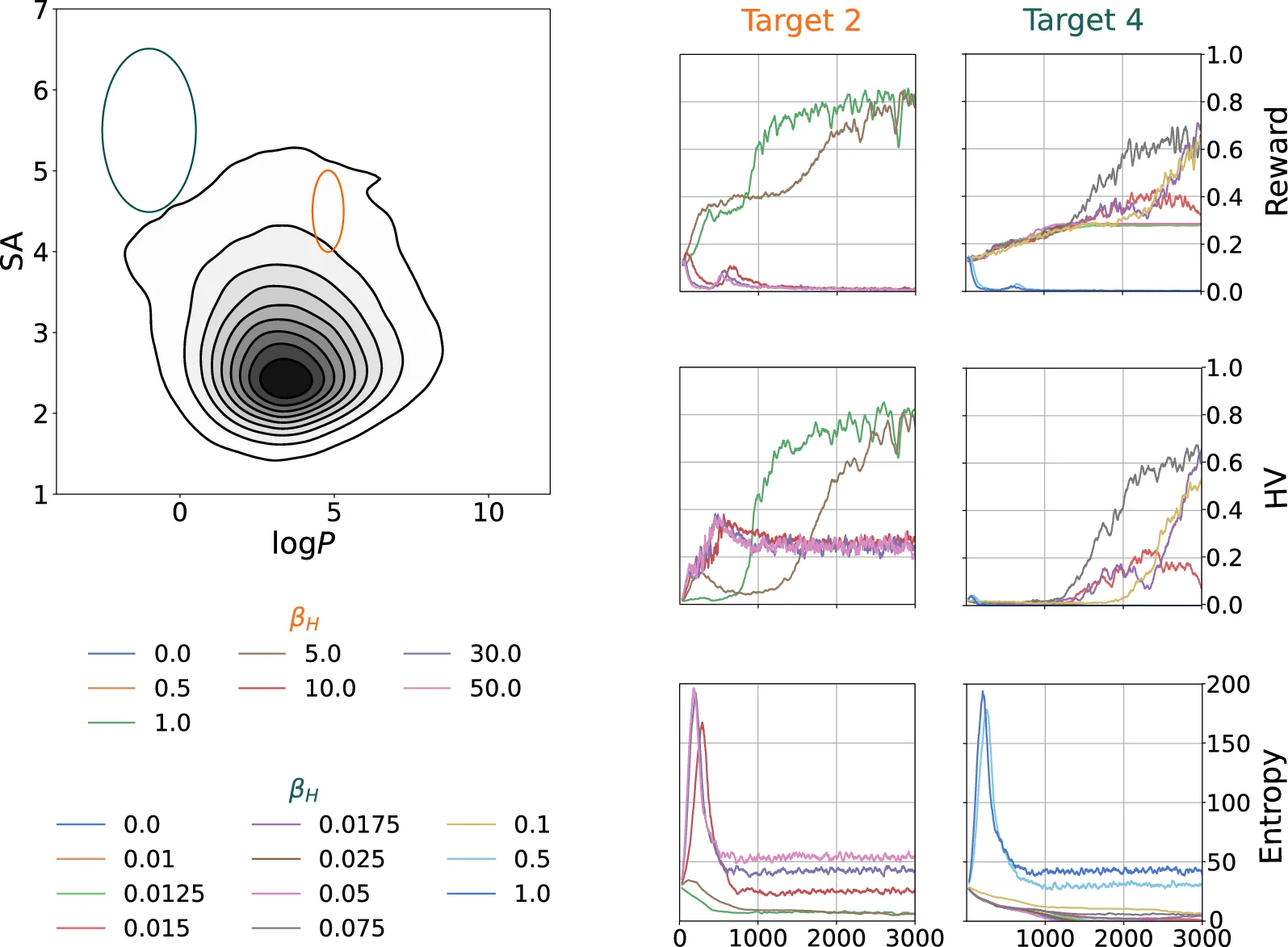
Effect of entropy regularization on sum scalarization with static weights. Left: (log*P*, SA) target distributions (Target 2, blue and Target 4, green) relative to the prior. Right: training dynamics for the two target regions (Target 2, orange, left column and Target 4, green, right column), showing reward, hypervolume (HV), and batch entropy (rows) over training epochs. Within each panel, curves correspond to different values of $\beta _H$, including the baseline $\beta _H=0$

We next investigated whether explicit entropy regularization can mitigate the mode-collapse behavior observed in challenging regimes. Unlike weight scheduling, which adapts the objective weights over training, entropy regularization instead biases the policy toward sustained exploration. Concretely, we augmented the RL objective with an entropy bonus scaled by $\beta _H$ (section Entropy regularization), with the goal of preventing the collapse phenomena reported in section Impact of scalarization.

To isolate the effect of entropy, we focused exclusively on the setting that exhibited the strongest instability in earlier experiments: sum scalarization with static weights. We evaluated two regimes: Target 2, which is relatively far to the prior with a narrow desirability region, and an additional, substantially farther target (Target 4; see Table S1 for alignment metrics). This design allows us to probe how the stability of entropy regularization depends on the tightness of the target region and on prior support. In both cases the $\beta _H=0$ serves as baseline case (no entropy regularization) which results in model collapse.

Figure 7 summarizes the impact of varying $\beta _H$. The left panel shows the joint distribution of generated molecules in (log*P*, SA) space, including a visual reference to the target regions, while the right panels report the corresponding training dynamics (reward, HV, and entropy). In both regimes, the baseline case $\beta _H = 0$ (no entropy regularization) leads to collapse, consistent with the failure mode identified previously.

For Target 4, entropy regularization prevents collapse across a comparatively broad range of values. In particular, $\beta _H \in \{0.5, 1, 5\}$ yields stable training. Among these, $\beta _H = 1$ provides the strongest overall performance, reaching the highest reward (approximately 0.75) and the highest HV. The trajectories for $\beta _H = 1$ and $\beta _H = 5$ converge to similar values by around epoch 2800. Increasing $\beta _H$ beyond 5 degrades performance: for $\beta _H \in \{10, 30, 50\}$, training converges to a low HV of approximately 0.25 by epoch 1000.

Notably, very large $\beta _H$ values induce strong early instabilities. For $\beta _H \in \{10, 30, 50\}$, entropy spikes to extreme values and then decays, converging to a stable value. In contrast, the stable settings maintain markedly lower peak entropy. This behavior is consistent with an over-regularized regime in which the entropy term dominates the learning signal early on, pushing the policy toward near-uniform sampling and preventing recovery of a high-reward, target-aligned distribution.

Target 2 proved substantially more difficult to stabilize and required a finer sweep over $\beta _H$. Larger values did not prevent collapse, motivating a search toward lower values. Very small values (e.g., $\beta _H = 0.0150$ or 0.0175) also were insufficient, resulting in collapse for the majority of random seeds. In our sweep, only $\beta _H \in \{0.075, 0.1\}$ consistently maintained stability. This indicates that Target 2 is highly sensitive to the exploration-exploitation balance, likely because the desirability region is narrow: the policy must preserve enough exploration to avoid premature concentration on an imbalanced ridge, yet still concentrate sufficiently to satisfy the tight constraints.

Across both regimes, we observe a clear “sweet spot” in $\beta _H$ where entropy regularization prevents collapse while preserving optimization progress. While entropy bonuses are a standard tool in RL, our results highlight a practical sensitivity in multi-objective molecular generation: the stabilizing range of $\beta _H$ depends strongly on the effective sparsity and tightness of the target region. For Target 4 (far but weakly constrained), extended exploration remains compatible with convergence, yielding a relatively wide interval of stabilizing $\beta _H$ values. For Target 2 (far and narrow), the stable region is much tighter in our experiments ($\beta _H \in [0.075, 0.1]$): smaller values are insufficient to prevent early over-concentration, whereas larger values over-regularize the policy and hinder convergence toward the narrow optimum. Thus, entropy regularization can act as an effective complementary mechanism against collapse, but only under careful tuning, which effectively increases the complexity of using this approach.

### Three-objective optimization

We next investigate a three-objective optimization setting to assess whether the observed scalarization-dependent trends persist as the dimensionality of the objective space increases. To this end, we add molecular weight (MW) as a third property to be optimized alongside log*P* and SA.

**Fig. 8:**
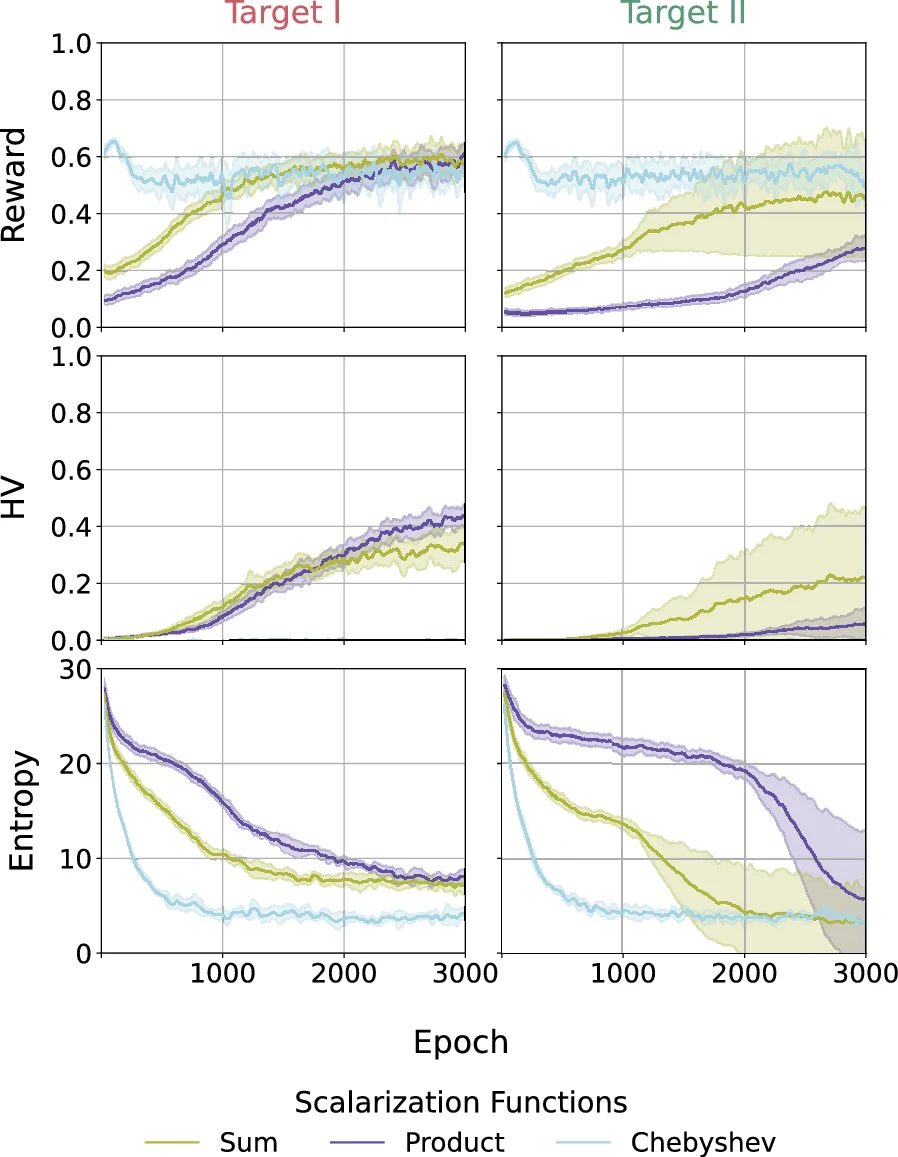
Training progression statistics for the two target regions. The left panel illustrates the KDE of the retaining data of the prior model against the targets (indicated by red and green areas). The right panel details the evolution of average reward (top row), hypervolume (middle row) and token-level entropy (bottom row) over the course of fine-tuning

We evaluate two narrow target regions: one located close to the prior distribution and one positioned further away. To distinguish these targets from the previously analyzed ones, we denote them as Target I and Target II, using Roman numerals. To keep the comparison controlled, the target regions differ only in their SA value, while the log*P* and MW targets are kept fixed (see Table S2 in the Supplementary Information). We analyze the evolution of reward, hypervolume (HV), and policy entropy during RL fine-tuning, with the corresponding results shown in Fig. 8.

#### Impact of scalarization

***Target-wise training dynamics***

Target I defines a narrow desirability region close to the prior distribution. Across scalarization methods, the average reward converges to a similar value of approximately 0.5. The reward value is substantially lower than the rewards observed in the corresponding two-objective setting, highlighting the more restrictive optimization problem.

For sum and product scalarization, the average reward converges steadily over training, with sum scalarization reaching the stable regime faster than product. In contrast, Chebyshev scalarization shows no significant improvement over the entire run, indicating limited progress toward the target region.

The hypervolume trajectories increase for both sum and product scalarization, with product scalarization reaching a higher final HV value. This suggests that, despite achieving a comparable final average reward, product scalarization maintains a more favorable distribution of trade-offs across the three objectives. By contrast, the HV of Chebyshev scalarization remains close to zero throughout training, indicating that the generated molecules rarely approach the target region in a way that contributes meaningfully to Pareto-front coverage.

The entropy trajectories further support these differences in optimization behavior. Under sum scalarization, policy entropy decreases steadily until approximately epoch 1300, indicating a gradual concentration of the policy. Product scalarization exhibits a pronounced entropy “shoulder” between epochs 300 and 1200, consistent with a longer exploration phase before convergence. Both sum and product scalarization eventually converge to similar entropy values. In contrast, Chebyshev scalarization shows a sharp early entropy decrease and reaches its final entropy level by approximately epoch 1000, consistent with premature policy concentration and limited subsequent exploration.

Target II is located further from the prior distribution while retaining the same width as Target I. Under both sum and product scalarization, the average reward increases slowly, with product scalarization performing sub-optimally regarding sum. With respect to Chebyshev, we observe a constant reward value, similar to Target I.

The hypervolume trajectories further confirm that all three scalarization methods face a difficult optimization regime. Product scalarization behaves more conservatively, with HV increasing only after approximately 2000 epochs. Sum scalarization reaches a higher average HV, but exhibits substantially larger variance across runs, suggesting that it can identify more favorable trade-off regions but does so less consistently. Chebyshev scalarization remains close to zero HV throughout training, indicating limited progress toward target-aligned trade-offs.

These HV trends are consistent with the entropy dynamics. The delayed HV increase under product scalarization coincides with a prolonged high-entropy phase, suggesting that the policy remains exploratory for longer before converging toward target-aligned regions. Sum scalarization shows a shorter exploratory phase and reaches higher HV earlier, but shows large run-to-run variability, implying a less conservative optimization behavior. Chebyshev scalarization behaves similarly to Target I, with limited reward and HV improvement together with an early entropy decrease.

***Diversity analysis***

To further characterize the effect of scalarization for Target I and II, we analysed the evolution of the generated chemical space through pairwise Tanimoto-similarity distributions of molecules generated at different training checkpoints.

**Fig. 9:**
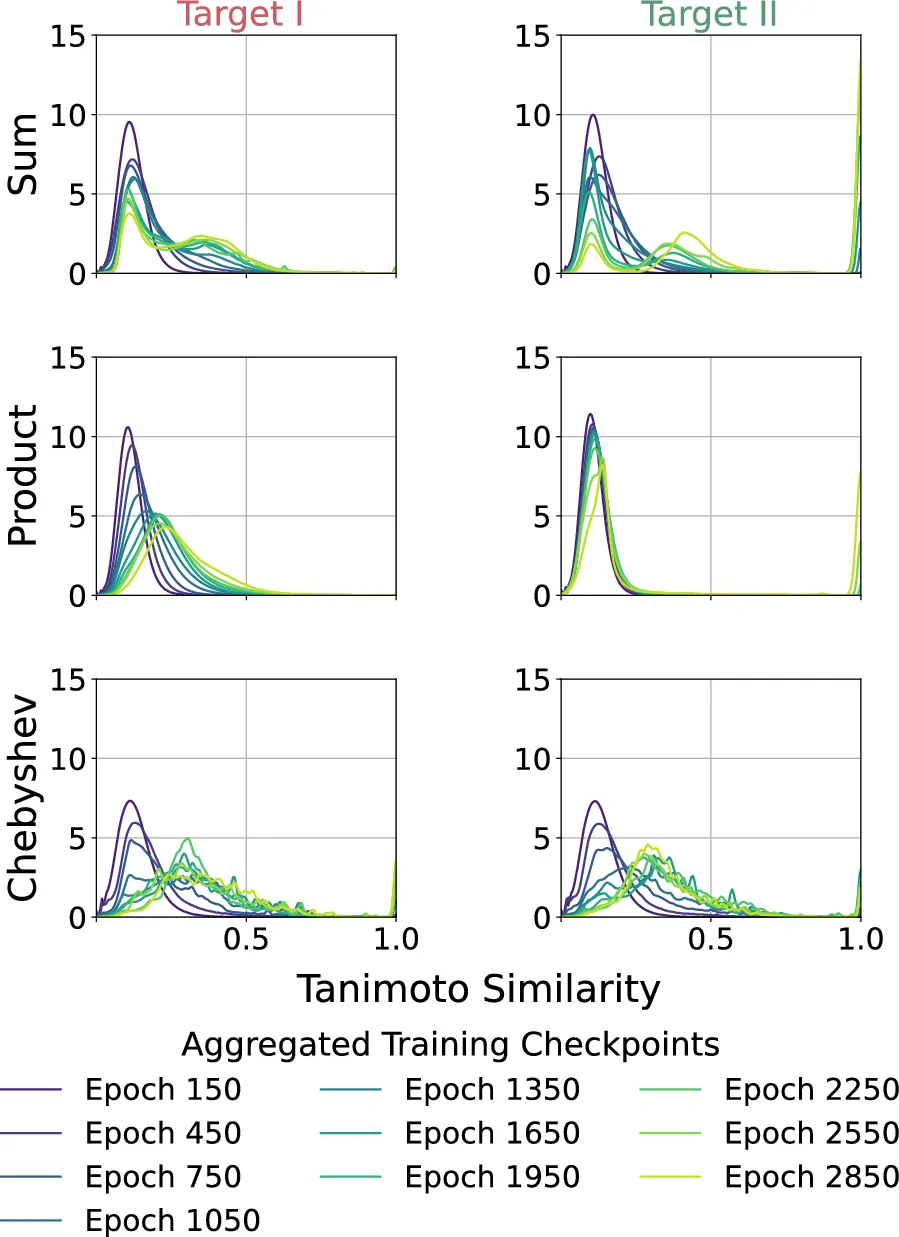
Evolution of Tanimoto similarity distributions under RL fine-tuning for an averaged run. Each panel shows the KDE of the Tanimoto similarity distributions for a target region (columns) across multiple training checkpoints (color-coded by epoch). Comparisons are shown across three scalarization methods: sum (top), product (middle), and Chebyshev (bottom)

For Target I, both sum and product scalarization maintain mostly low-to-moderate Tanimoto similarities throughout training, indicating that neither method collapses immediately to a single highly redundant molecular family. However, the two methods differ in how the generated distribution evolves. Under sum scalarization, the initial low-similarity density is retained while an additional mode at intermediate similarity emerges over training. This suggests that the policy continues to generate molecules from the initially accessible region while also learning a second, more target-aligned structural region. In contrast, product scalarization shows a more coherent shift of the distribution toward intermediate similarities, rather than the coexistence of two clearly separated density regions (Fig. 9).

This difference is consistent with the selection pressure induced by the scalarization functions. Sum scalarization is permissive toward partial objective satisfaction: molecules that satisfy only a subset of the objectives can still receive intermediate reward. As a result, the policy can retain probability mass in partially target-aligned regions while also discovering new higher-reward structures. Product scalarization, by contrast, suppresses partial solutions because a low desirability value in any single objective strongly reduces the scalarized reward. This creates stronger pressure to move the generated distribution toward regions where the objectives improve jointly, leading to a smoother migration of density rather than the simultaneous maintenance of distinct molecular populations.

For Target II, the same qualitative pattern becomes more pronounced. Under sum scalarization, the Tanimoto distribution develops a clear late-training density at high similarity, including a peak close to similarity 1. This indicates that, once the policy identifies a rewarding region, it can concentrate strongly around a narrow set of highly similar molecules. Thus, the higher reward and HV observed for sum scalarization in this target come with an increased tendency toward structural redundancy. Product scalarization, in contrast, maintains lower similarity values for a longer portion of training, consistent with its extended exploration phase and slower reward increase. However, this sustained diversity is accompanied by lower reward and HV, indicating that product scalarization preserves exploration but struggles to exploit the narrow target-aligned region efficiently in the three-objective setting.

Chebyshev scalarization exhibits broader and less directed similarity distributions across both targets. However, this apparent diversity does not correspond to effective optimization. Together with the near-zero HV and limited reward improvement observed in the training curves, the Tanimoto distributions indicate that Chebyshev scalarization does not guide the policy toward a useful target-aligned trade-off region. Thus, the generated molecules may remain structurally dispersed, but this diversity is not productive in terms of multi-objective optimization.

Overall, the three-objective experiments show that increasing the dimensionality of the objective space amplifies the optimization difficulties already observed in the two-objective setting. As in the two-objective case, product scalarization maintains a more conservative and exploratory optimization trajectory, while sum scalarization tends to exploit single target-aligned regions more rapidly. Sum scalarization tends to preserve partially rewarded regions while also developing additional high-similarity modes, reflecting its ability to exploit partial objective satisfaction. Product scalarization produces a more coherent but slower distributional shift, consistent with its stronger requirement for balanced improvement across all objectives.

#### Influence of weight scheduling strategies

We next investigate whether weight scheduling affects the three-objective optimization problem involving log*P*, SA, and MW. As in the two-objective setting, we compare multiple weight assignments while monitoring reward, hypervolume (HV), and policy entropy during RL fine-tuning.

For Target I, changes in weight scheduling strategies lead to broadly similar behavior. Across most scalarization methods, we observe no substantial differences in reward, HV, or entropy. Similarly to the 2-dimensional case, if the target is well supported by the prior, the optimization behavior is less dependent on the precise weight scheduling strategy. The main exception is the *cyclic* scheme, which yields lower HV values for sum and product scalarization, but slightly higher HV values for Chebyshev scalarization compared with the other weight scheduling strategies. Since Chebyshev scalarization already shows limited optimization performance in the three-objective setting, we interpret this improvement cautiously and do not consider it sufficient to alter the overall conclusion that Chebyshev is poorly aligned with this optimization problem. Detailed curves for Target I are provided in Figure S5.

**Fig. 10:**
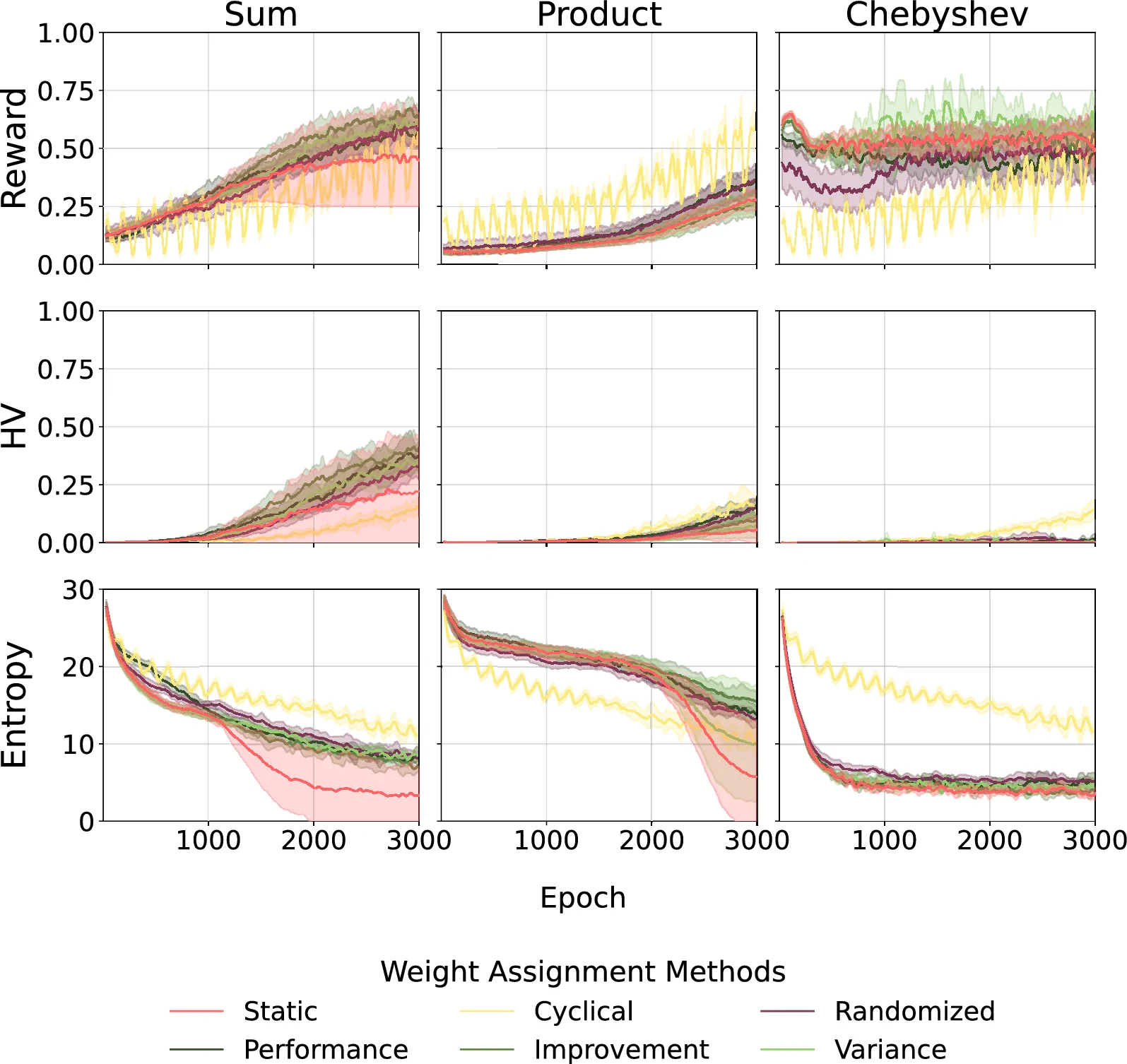
Effect of weight scheduling strategies on RL fine-tuning dynamics across scalarization methods for Target II. Columns correspond to scalarization functions (sum, product, Chebyshev), and rows report the evolution of average reward (top), hypervolume (middle), and batch entropy (bottom) over training epochs. Within each panel, curves for all weight schedules (*static*, *random*, *cyclical*, *performance*, *improvement*, *variance*) are overlaid to facilitate direct comparison

For Target II, the influence of weight scheduling becomes more pronounced (Fig. 10). Under sum scalarization, adaptive weight scheduling schemes, in particular *improvement*, outperform the *static* baseline and reach the highest reward and HV values. While the *static* baseline plateaus at an average reward around epoch 2000, the adaptive schemes continue to improve beyond this point. This suggests that dynamic reweighting can help the policy escape the limited optimization regime reached under fixed weights. The entropy trajectories provide additional insight into these differences. For sum scalarization, most weight scheduling strategies converge to similar final entropy values. However, the *static* scheme shows a transient entropy “shoulder” around epoch 1000, followed by a steady decrease until convergence around epoch 2000. This behavior coincides with the reward plateau and may indicate a temporary exploratory phase that is not sufficient to sustain further improvement. In contrast, the *cyclic* scheme maintains consistently higher entropy from approximately epoch 800 onward, suggesting prolonged exploration. Nevertheless, this additional exploration does not translate into the best reward or HV performance for sum scalarization, indicating that exploration alone is insufficient without an effective alignment of the weight schedule with the target region.

For product scalarization, we observe that the *cyclic* scheme achieves the highest average reward, while the *static* baseline performs worst. The cyclic scheme also reaches among the highest HV values, although the overall HV remains low compared with sum scalarization. This indicates that cyclic variation of the objective weights can be partially beneficial for product scalarization in the more challenging Target II setting, although not in a competitive manner. Its entropy remains among the lower trajectories, exceeded by most adaptive schemes and only clearly above the *static* baseline. This suggests that the benefit of cyclic weighting is not simply due to maintaining higher exploration, but rather to periodically shifting the optimization pressure across objectives. Thus, weight scheduling only partially mitigates the difficulty of this optimization target.

For Chebyshev scalarization, weight scheduling has only a limited effect on the overall optimization outcome. Although the average reward remains at an intermediate level across most scheduling strategies, the HV stays close to zero throughout training. This indicates that the scalarized reward signal does not translate into meaningful coverage of the target-aligned trade-off region. The entropy trajectories further suggest premature policy concentration: for most scheduling strategies, entropy rapidly decreases within the first few hundred epochs and then remains low. The *cyclic* scheme is the main exception, maintaining substantially higher and oscillatory entropy due to the periodic weight changes. However, this increased exploration does not lead to a corresponding improvement in HV. Thus, for this three-objective setting, the poor Chebyshev performance appears be roughly independent from the particular choice of weight scheduling strategy.

Overall, weight scheduling has limited impact for Target I, where the target region is close to the prior, but becomes more relevant for Target II, where the optimization problem is more difficult. In this regime, adaptive weighting improves sum scalarization, while cyclic weighting provides the strongest reward performance for product scalarization. However, none of the scheduling strategies fully overcomes the reduced reward and HV values observed in the three-objective setting. This indicates that weight scheduling can modulate the exploration-exploitation dynamics, but it does not remove the intrinsic difficulty of satisfying multiple simultaneous molecular objectives.

### Implications for higher-dimensional objective spaces

The successful optimization of multi-objective reward functions during RL fine-tuning depends on the interplay between two factors: the geometry of the scalarized reward landscape and the chemical-space distribution induced by the prior policy. The scalarization function determines how partial satisfaction of individual objectives is translated into a scalar learning signal, while the prior determines which regions of the reward landscape are initially accessible to the agent.

The different scalarization functions induce qualitatively distinct reward topographies. Sum scalarization creates ridges parallel to the coordinate axes, where molecules that satisfy only a subset of objectives can still obtain intermediate reward values. These ridges can provide a useful learning signal, but they can also become exploitable local optima when the policy improves one objective while neglecting the others. Product scalarization suppresses such ridges, since a near-zero desirability in any single objective drives the scalarized reward close to zero. This removes an easily exploitable partial-satisfaction region and can force the policy into a longer exploratory phase. Chebyshev scalarization behaves differently: due to its minimax structure, it can create broad regions with similar intermediate reward values, requiring the agent to explore until it reaches one of the comparatively small high-reward regions.

The comparison between the two- and three-objective settings shows that the relative advantage of product scalarization is dimension-dependent. In the two-objective experiments, product scalarization provided the most robust behavior because it discouraged solutions that optimized one objective at the expense of the other. However, in the three-objective setting, this strict coupling between objectives can become a limitation. If one objective remains poorly satisfied, the product reward is strongly suppressed, reducing the effective learning signal even when the policy improves along the remaining objectives. Sum scalarization is more permissive and can therefore maintain optimization progress in regimes where simultaneous improvement of all objectives is difficult. This explains why sum scalarization became more competitive in the three-objective experiments, particularly for the more challenging targets.

**Fig. 11:**
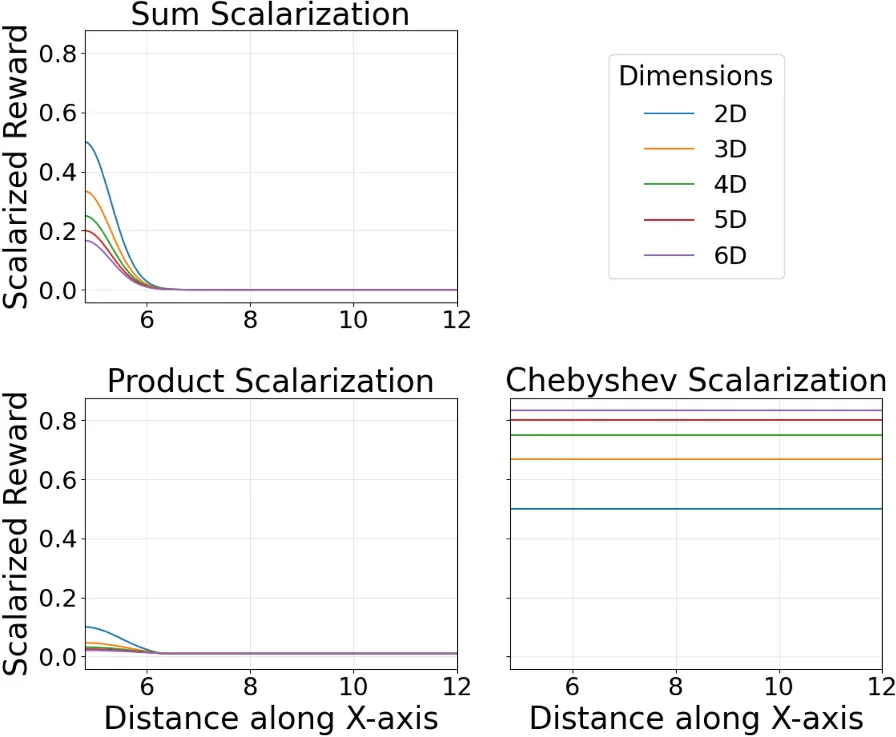
Dimensional decay of the ridges for each scalarization method, assuming $\sum _i^N w_i = 1$ with $w_i = \frac{1}{N}$

When extending this framework beyond three objectives, the geometry of the reward landscape changes systematically. This is illustrated by the dimensional decay of partial-satisfaction ridges in Fig. 11. The ridge shown corresponds to the region where one objective receives maximal desirability, i.e. a score of 1, while all remaining objective scores are 0. Under uniformly weighted sum scalarization, the height of this ridge scales as 1/*N*, where *N* is the number of objectives. Thus, in a two-objective problem, such a ridge still yields a relatively high reward of 0.5, whereas in a six-objective problem the corresponding reward decreases to approximately 0.17. This dimensional decay suggests that sum scalarization may become less prone to ridge-induced collapse as the number of objectives increases, consistent with the weaker ridge-dominated behavior observed when comparing the two-objective Target 2 experiments with the three-objective setting.

However, increasing the number of objectives introduces new difficulties. For product scalarization, the high-reward region becomes increasingly sparse as dimensionality increases. Assuming Gaussian desirability functions with fixed widths, simultaneous high reward requires all objectives to fall within their respective target regions. Although the width of each individual desirability function remains unchanged, the joint volume in which all desirabilities are high decreases rapidly with the number of objectives. As a result, the product reward landscape becomes increasingly sparse, which can weaken the learning signal and hinder RL fine-tuning. For Chebyshev scalarization, the opposite issue can arise. Under normalized weights, the penalty associated with any single poorly optimized objective decreases as the number of objectives increases. Consequently, broad regions of the landscape can receive relatively high scalarized reward even when most objectives are poorly satisfied. In the limit of many objectives, this risks creating a weakly informative plateau in which the agent receives a high reward for molecules that do not meaningfully satisfy the full multi-objective target.

Thus, higher-dimensional objective spaces may reduce ridge-induced collapse for sum scalarization, but they can also make product scalarization increasingly sparse and Chebyshev scalarization increasingly insensitive.

### Limitations and future work

This study was designed to isolate the effect of reward scalarization under a fixed RL fine-tuning setup. Several limitations should therefore be considered when interpreting the results. First, all experiments use a fixed 3-layer LSTM generator. Since the scalarization strategies studied here operate at the level of reward aggregation, they are not specific to recurrent architectures. Nevertheless, attention-based architectures may affect absolute performance, exploration behavior, sample efficiency, molecular validity, diversity, and the severity of optimization instabilities. Prior work suggests that the relative suitability of RNNs and Transformers in molecular language modelling depends on the molecular regime, with RNNs competitive for local-feature-dominated tasks and Transformers more advantageous for larger molecules or longer sequences where global dependencies are more important [45]. Since our experiments focus on small-molecule generation with a maximum sequence length of 120 tokens, we consider the LSTM a suitable controlled policy for this study. A systematic evaluation of the observed scalarization trends with Transformer backbones remains an important direction for future work.

Second, all experiments were performed under a common memory kernel to discourage repeated generation of structurally similar molecules. The kernel is scalarization-independent in its definition, since it depends only on Tanimoto similarity to previously generated molecules and is applied uniformly across methods. However, its effective impact may still be scalarization-dependent, because different scalarization strategies induce different molecular distributions and therefore different frequencies of similarity-based reward suppression. Thus, the reported results should be interpreted as the behavior of each scalarization method under a common diversity constraint, rather than as scalarization behavior in the absence of diversity filtering. Future work should include sensitivity analyses over memory-kernel thresholds or ablations without the kernel.

Third, the present experiments consider unconstrained de novo generation. In ligand-based lead optimization, it is often desirable to restrict the search to molecules containing predefined scaffolds or chemotypes. Such scaffold constraints are directly relevant for focused library design, scaffold decoration, linker design, and R-group replacement, but would also alter the feasible chemical space and could change the observed diversity, novelty, and Pareto-front structure. We therefore consider scaffold-constrained multi-objective RL an important future direction for evaluating whether the scalarization trends observed here persist under ligand-based design constraints.

Finally, the higher-dimensional analysis presented in section Implications for higher-dimensional objective spaces suggests that scalarization behavior may change substantially as the number of objectives increases: sum-scalarization ridges decay with dimensionality, product rewards become increasingly sparse, and Chebyshev-type rewards may become less informative. Extending the empirical analysis to larger objective sets, non-Gaussian desirability functions, different priors, and other RL algorithms would help determine how general these trends are.

## Conclusion

This work examined a consequential design choice in RL fine-tuning for de novo molecular generation: how multi-objective goals are converted into a scalar training signal, and how this conversion affects optimization stability. Using a REINVENT loss, we analyzed the interplay between scalarization functions, weight scheduling, and entropy regularization in a two- and three-objective settings, tracking reward, hypervolume (HV), policy entropy and structural diversity to capture both optimization progress and diversity collapse.

Across experiments, optimization difficulty was primarily dominated by two factors: the constraints in the desirability region and the degree of prior support for the target region. Broad and/or well-supported targets allowed multiple optimization paths, such that methodological differences had limited impact. In contrast, narrow targets in low-support regions produced sparse reward landscapes in which algorithmic choices that may appear interchangeable became decisive for stability and coverage of the trade-off surface.

The results show that scalarization is a central determinant of optimization stability and trade-off coverage. In two-objective settings, product scalarization was the most robust strategy in difficult regimes. By suppressing partially optimized ridge solutions, it reduced the risk of stagnation and collapse observed under sum scalarization. However, the three-objective experiments revealed that this advantage is dimension-dependent. As the number of objectives increases, the strict coupling imposed by product scalarization can weaken the learning signal when one objective remains poorly satisfied. In this regime, sum scalarization becomes more competitive because it can still reward partial progress, although this permissiveness may also promote structural redundancy.

The diversity analysis further showed that reward and HV alone are insufficient to characterize RL fine-tuning behavior. Sum scalarization can achieve higher reward or HV while retaining multiple structural populations, including highly similar molecules. Product scalarization instead induces a more coherent but slower redistribution of probability mass in chemical space, consistent with its stronger requirement for balanced objective improvement. Chebyshev scalarization showed limited target-aligned optimization in the tested settings, with low HV and poorly directed diversity. These considerations suggest that higher-dimensional multi-objective molecular optimization will require scalarization strategies that balance learnability, target alignment, and diversity preservation.

Stabilization strategies modulated these effects but did not eliminate the underlying dependence on target geometry and scalarization. Dynamic weighting improved optimization in difficult regimes, but the most effective strategy depended on the scalarization function and target location. Entropy regularization provided an additional safeguard against collapse, but only within a target-dependent range: insufficient regularization failed to prevent premature concentration, whereas excessive regularization hindered convergence.

Together, these findings suggest practical guidelines for constrained MPO fine-tuning. First, scalarization should be treated as a first-class design choice rather than a technical detail. Product scalarization is advantageous when balanced objective satisfaction is achievable and ridge-induced collapse is a concern, but it may become too restrictive in higher-dimensional or weakly supported targets. Sum scalarization can be useful when partial progress is needed to maintain a learning signal, but it should be monitored carefully for objective dominance and structural redundancy. Chebyshev scalarization, in the tested settings, was not sufficient to produce reliable target-aligned optimization. Second, dynamic weighting and entropy regularization can improve stability, but their benefit depends on the target geometry, prior support, and scalarization function. Third, optimization performance should be evaluated jointly through reward, HV, entropy, and structural diversity, since each metric captures a different aspect of the fine-tuning dynamics.

In conclusion, RL fine-tuning stability is shaped not only by the model class, but critically by how objectives are scalarized and regularized relative to target constraints and prior support. Broad targets can mask methodological differences, whereas narrow and weakly supported objectives expose sharp failure modes; robust scalarization and stabilization mechanisms are therefore essential for reliable multi-objective molecular optimization.

## Supplementary Information

Supplementary material 1.

## Data Availability

Accompanying code for this work can be found at "https://github.com/palominohernandez/ExploRL25". Supplementary information is available with details of pretraining, token information, other details on RL-based fine-tuning.
