# Supplementary material for: The impact of reward scalarization and weight scheduling on optimization dynamics in multi-objective molecular design
Source: J Cheminform. 2026 Aug 19;18:118. doi: 10.1186/s13321-026-01273-8 (PMC13491586; doi:10.1186/s13321-026-01273-8)
Supplement: Supplementary file 1 [file 13321_2026_1273_MOESM1_ESM.pdf]

The Impact of Reward Scalarization and Weight Scheduling on Optimization Dynamics in Multi-Objective Molecular Design

Lucas Leuschner[1†] and Oscar Palomino-Hernandez[1,2*†]

[1]Department of Chemistry, Johannes Gutenberg University Mainz, Duesbergweg 10-14, D-55128 Mainz, Germany.
[2]Institute for Quantitative and Computational Biosciences (IQCB), Johannes Gutenberg University Mainz, Johannes-von-Müller-Weg 6, D-55128 Mainz, Germany.

*Corresponding author(s). E-mail(s): opalomin@uni-mainz.de;
Contributing authors: luleusch@uni-mainz.de;
†These authors contributed equally to this work.

Supporting Information

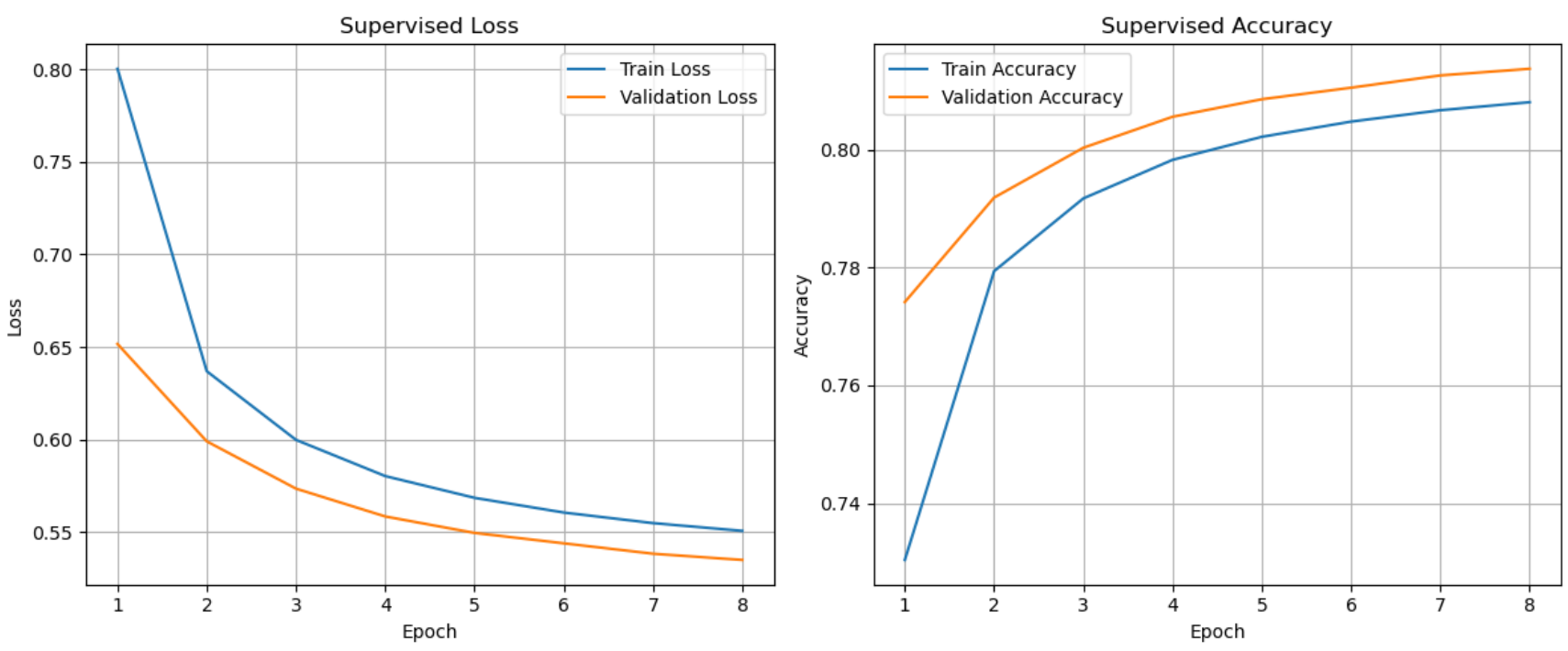

Figure S1 Training curves of the prior model pretrained on the ChEMBL33 dataset.

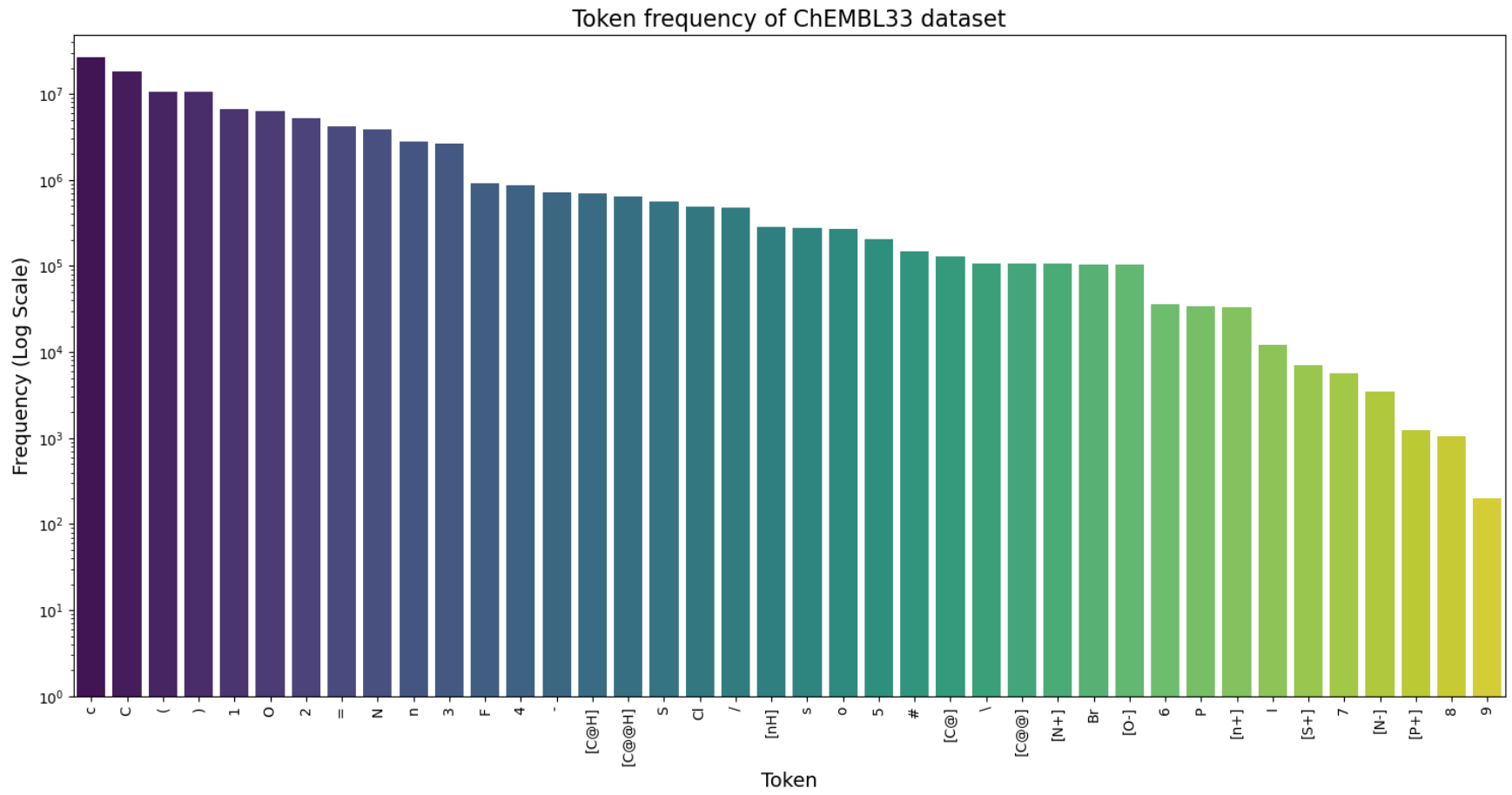

Figure S2 Frequency of tokens used to encode the ChEMBL33 dataset.

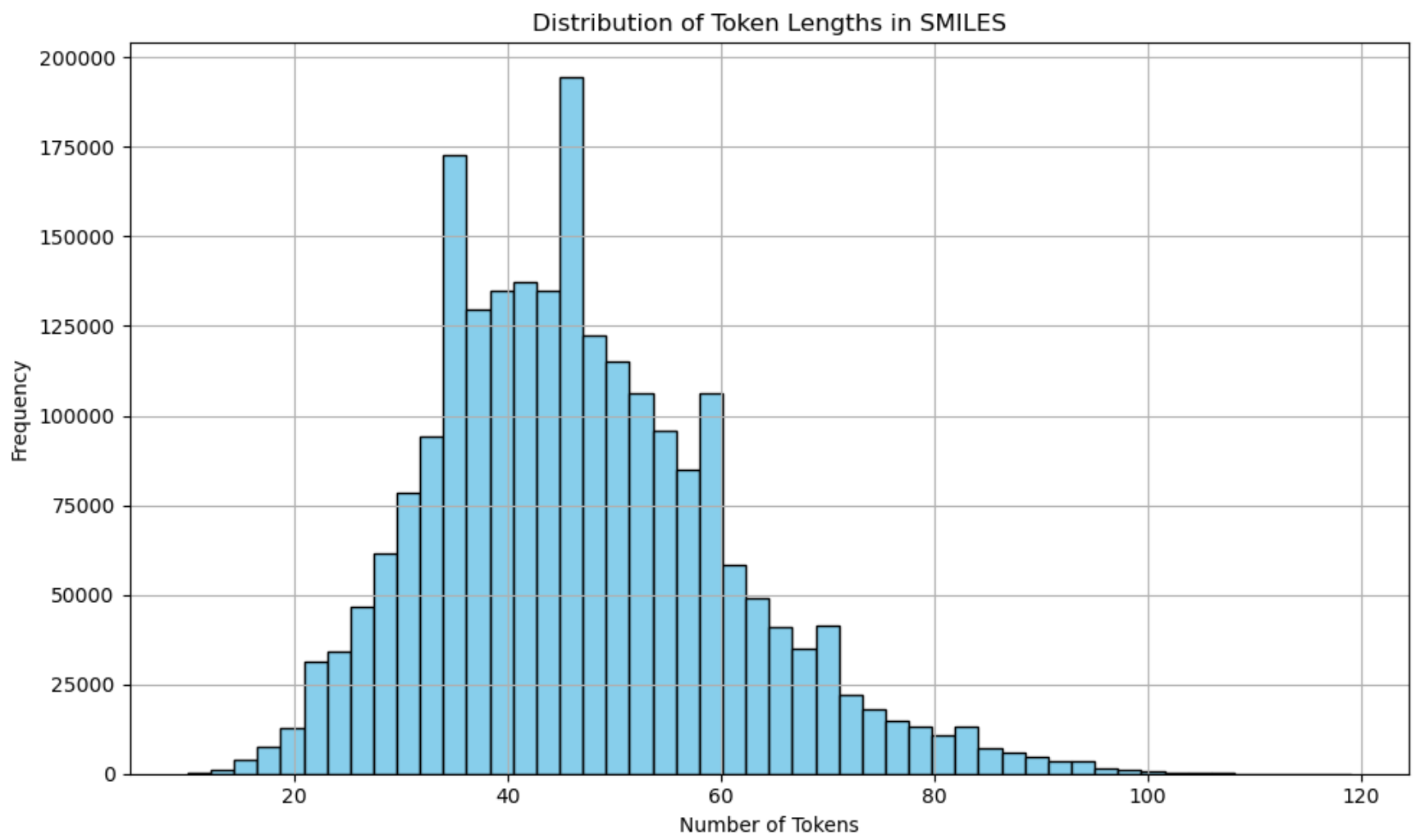

Figure S3 Distribution of token length of the molecules in the ChEMBL33 dataset.

Table 1 Prior distribution and target desirability regions in (log$P$, SA) space, together with prior–target alignment metrics. Prior density at target refers to the estimated prior density evaluated at the target mean in property space.

	log$P$		SA		Average Wasserstein dist. to prior	Prior density at target	Mahalanobis dist. to prior
Region	μ	σ	μ	σ			
Prior	3.4	1.9	2.9	0.8	–	–	–
Target 1	4.8	0.5	3.5	0.5	1.107	3.71×10^{-2}	1.063
Target 2	4.8	0.5	4.5	0.5	1.575	9.56×10^{-3}	2.131
Target 3	6.0	2.0	4.5	1.0	2.083	4.94×10^{-3}	2.467
Target 4	-1.0	1.5	5.5	1.0	3.516	2.36×10^{-4}	3.947

Table 2 Prior distribution and target desirability regions in (log P, SA, MW) space, together with prior–target alignment metrics. Prior density at target refers to the estimated prior density evaluated at the target mean in property space.

Region	log P μ	log P σ	SA μ	SA σ	MW μ	MW σ	Average Wasserstein dist. to prior	Prior density at target	Mahalanobis dist. to prior
Prior	3.4	1.9	2.9	0.8	–	–	–	–	–
Target I	4.8	0.5	3.5	0.5	550	50	52.848	1.189×10^{-4}	1.475
Target II	4.8	0.5	4.5	0.5	550	50	53.154	1.887×10^{-5}	2.166

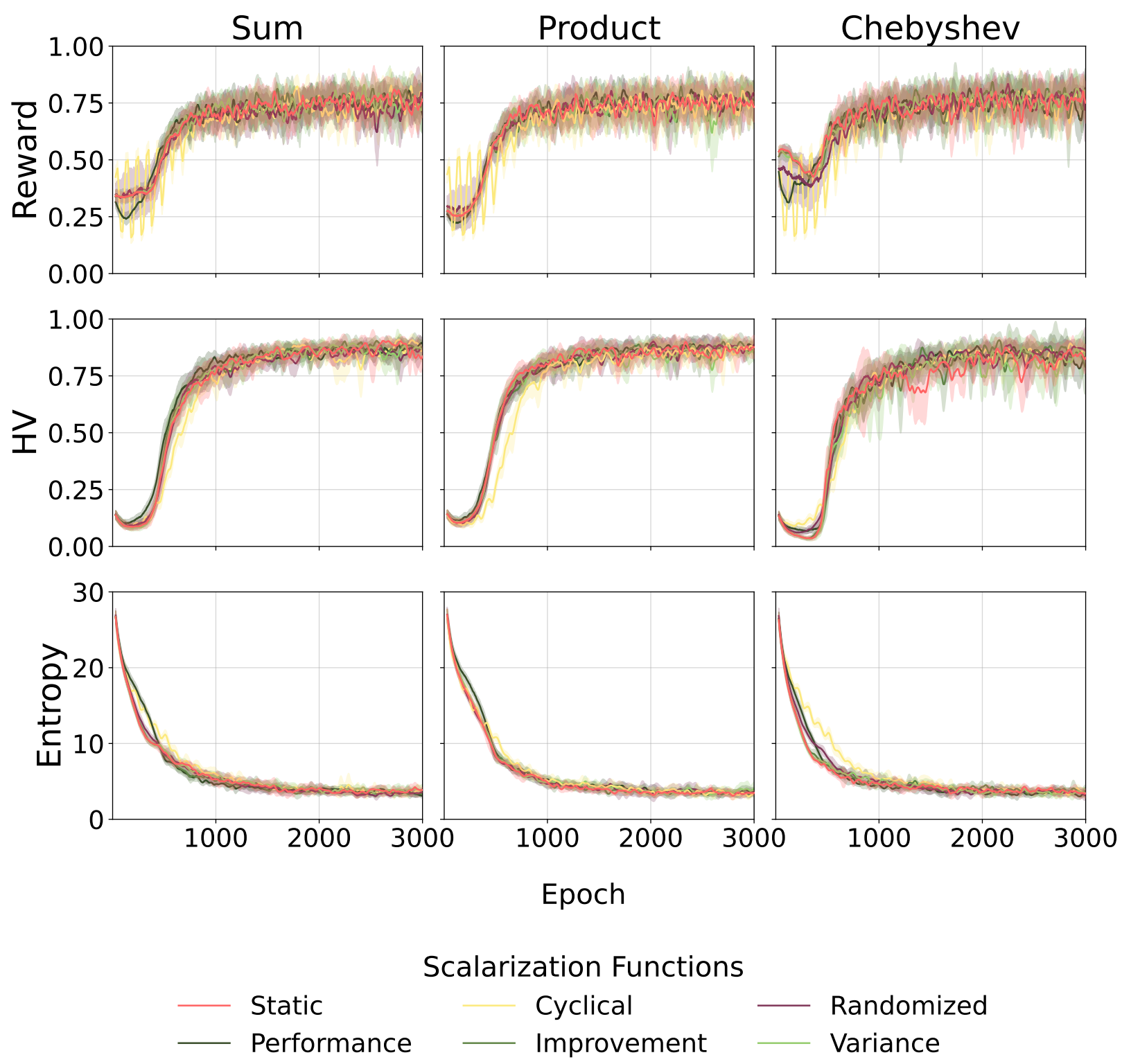

Figure S4 Effect of weight scheduling strategies on RL fine-tuning dynamics across scalarization methods for Target 3. Columns correspond to scalarization functions (sum, product, Chebyshev), and rows report the evolution of average reward (top), hypervolume (middle), and batch entropy (bottom) over training epochs. Within each panel, curves for all weight schedules (static, random, cyclical, performance, improvement, variance) are overlaid to facilitate direct comparison.

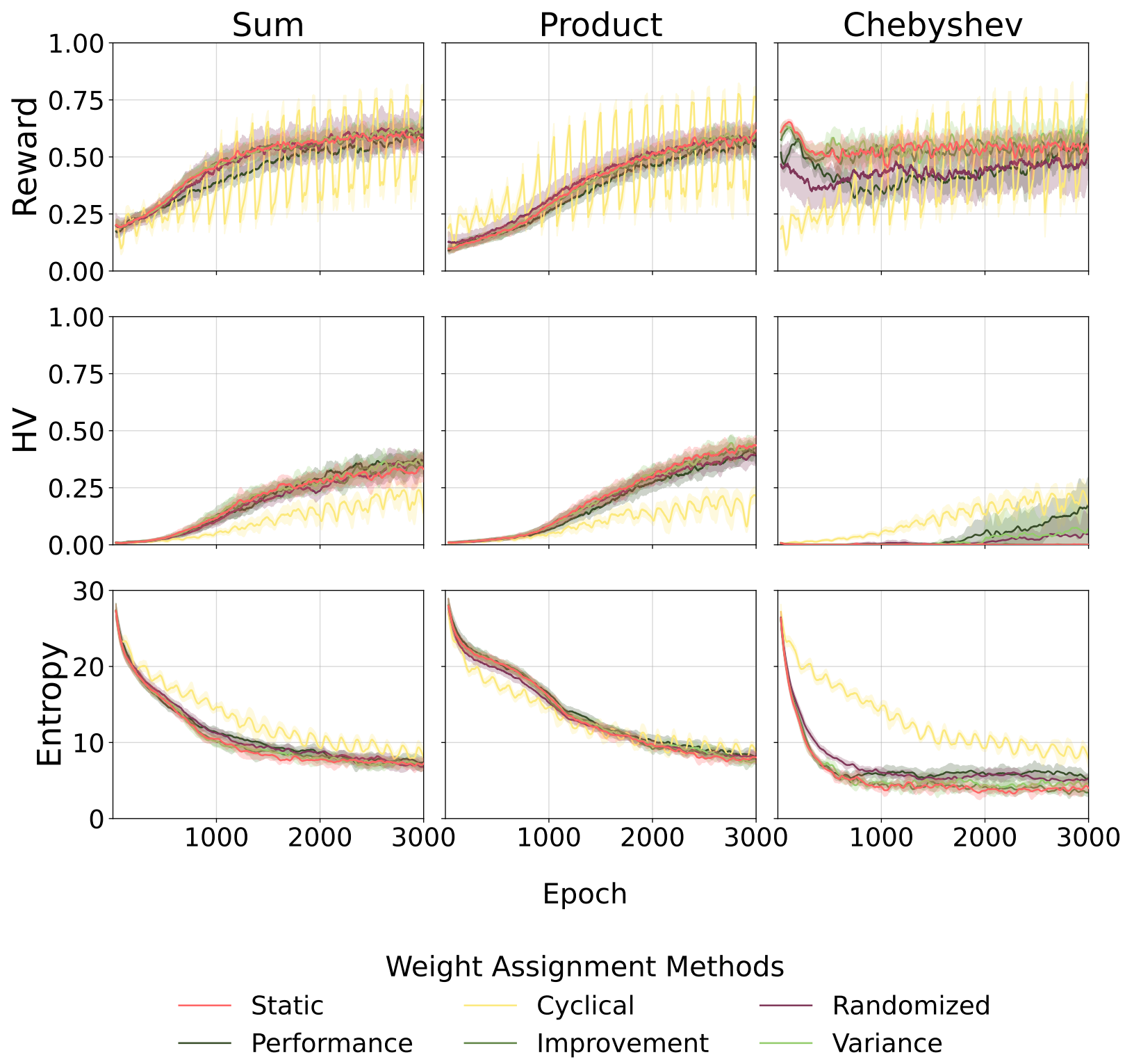

Figure S5 Effect of weight scheduling strategies on RL fine-tuning dynamics across scalarization methods for Target I. Columns correspond to scalarization functions (sum, product, Chebyshev), and rows report the evolution of average reward (top), hypervolume (middle), and batch entropy (bottom) over training epochs. Within each panel, curves for all weight schedules (static, random, cyclical, performance, improvement, variance) are overlaid to facilitate direct comparison.

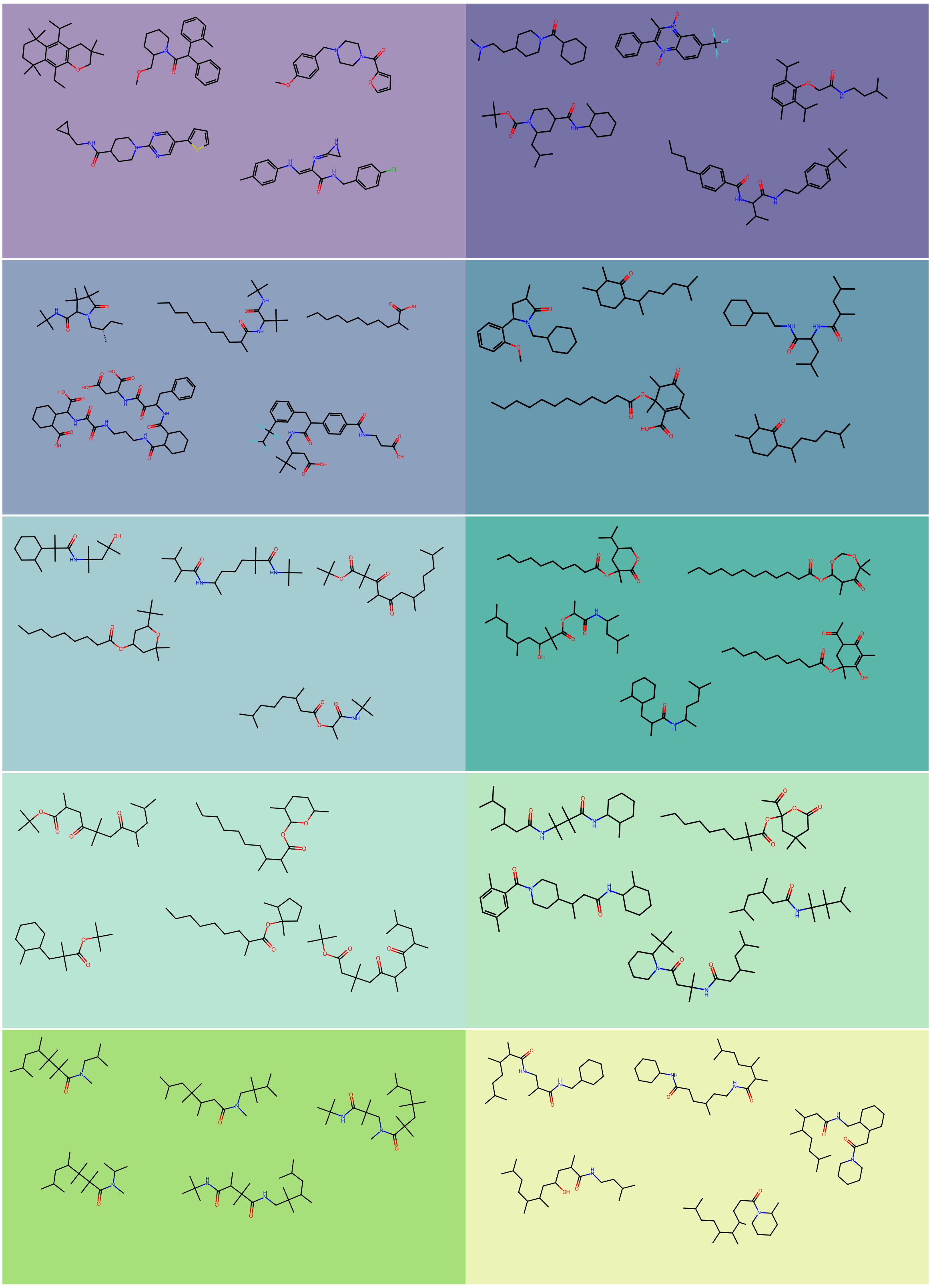

Figure S6 Molecules sampled from checkpoints for a agent trained with sum scalarization and static weight assignment to optimize for target 1. Epochs are ascending from left to right, top to bottom starting at epoch 150 up to 2850 in 150 intervals.

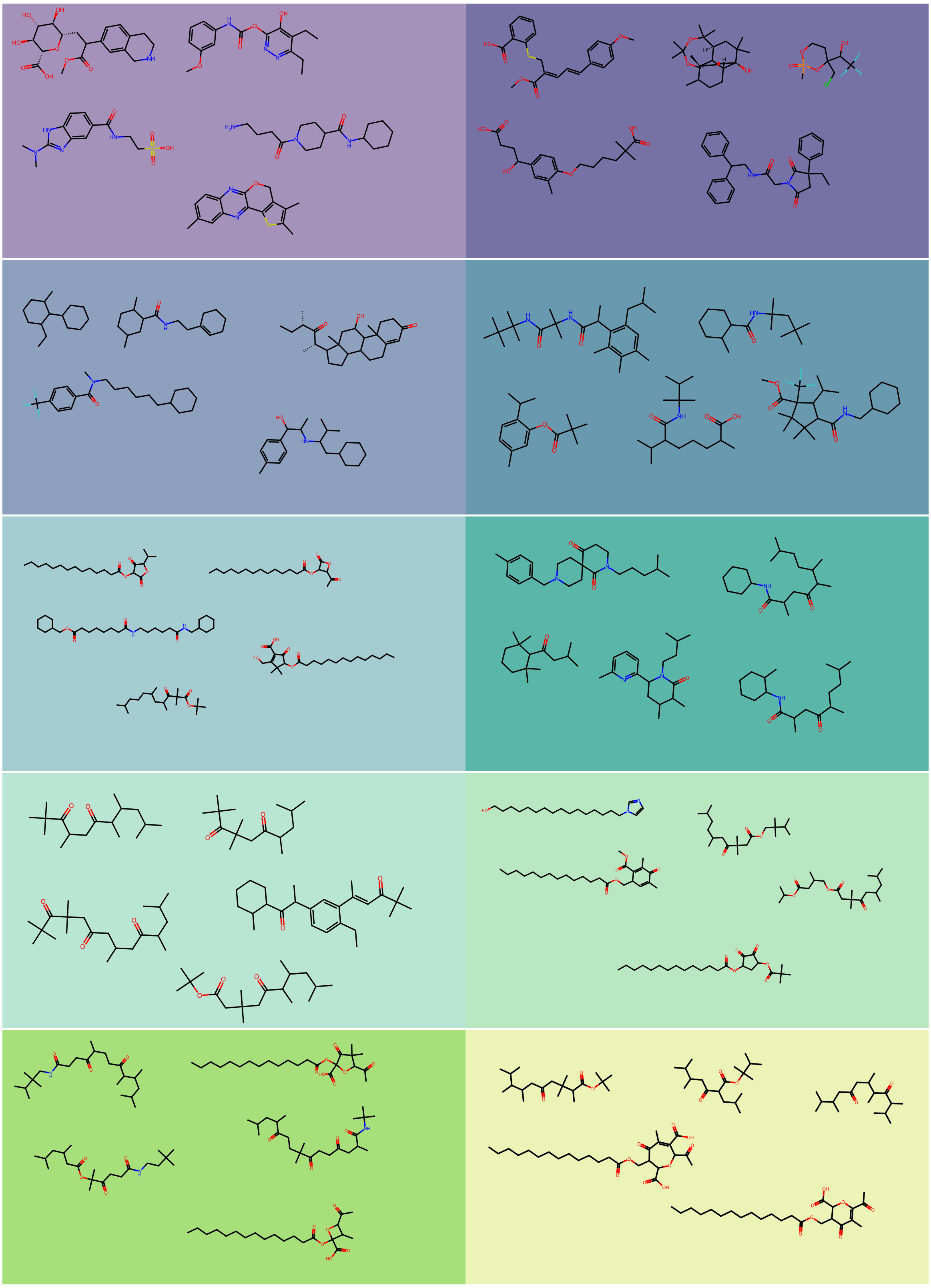

Figure S7 Molecules sampled from checkpoints for a agent trained with product scalarization and static weight assignment to optimize for target 1. Epochs are ascending from left to right, top to bottom starting at epoch 150 up to 2850 in 150 intervals.

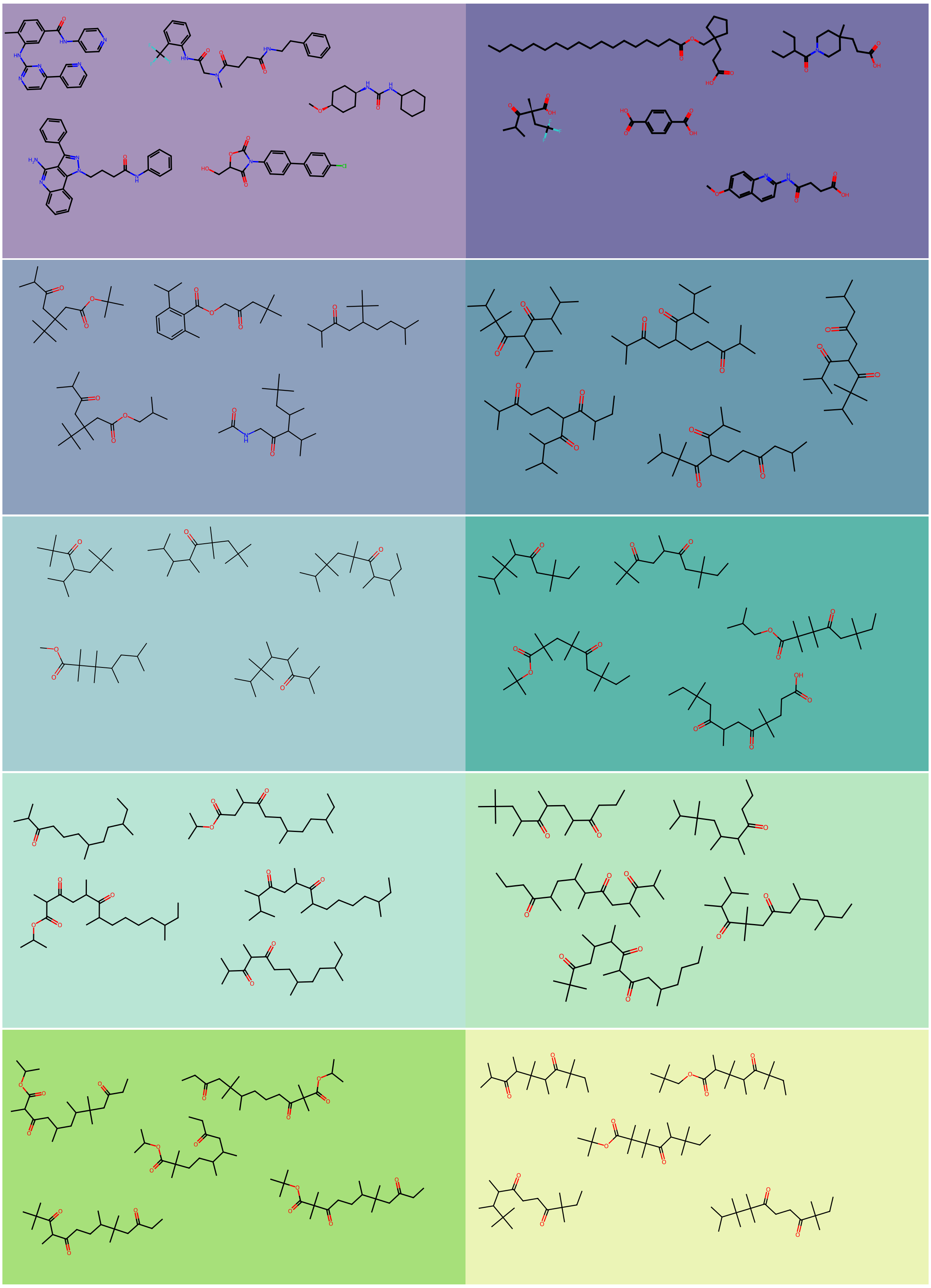

Figure S8 Molecules sampled from checkpoints for a agent trained with Chebyshev scalarization and static weight assignment to optimize for target 1. Epochs are ascending from left to right, top to bottom starting at epoch 150 up to 2850 in 150 intervals.

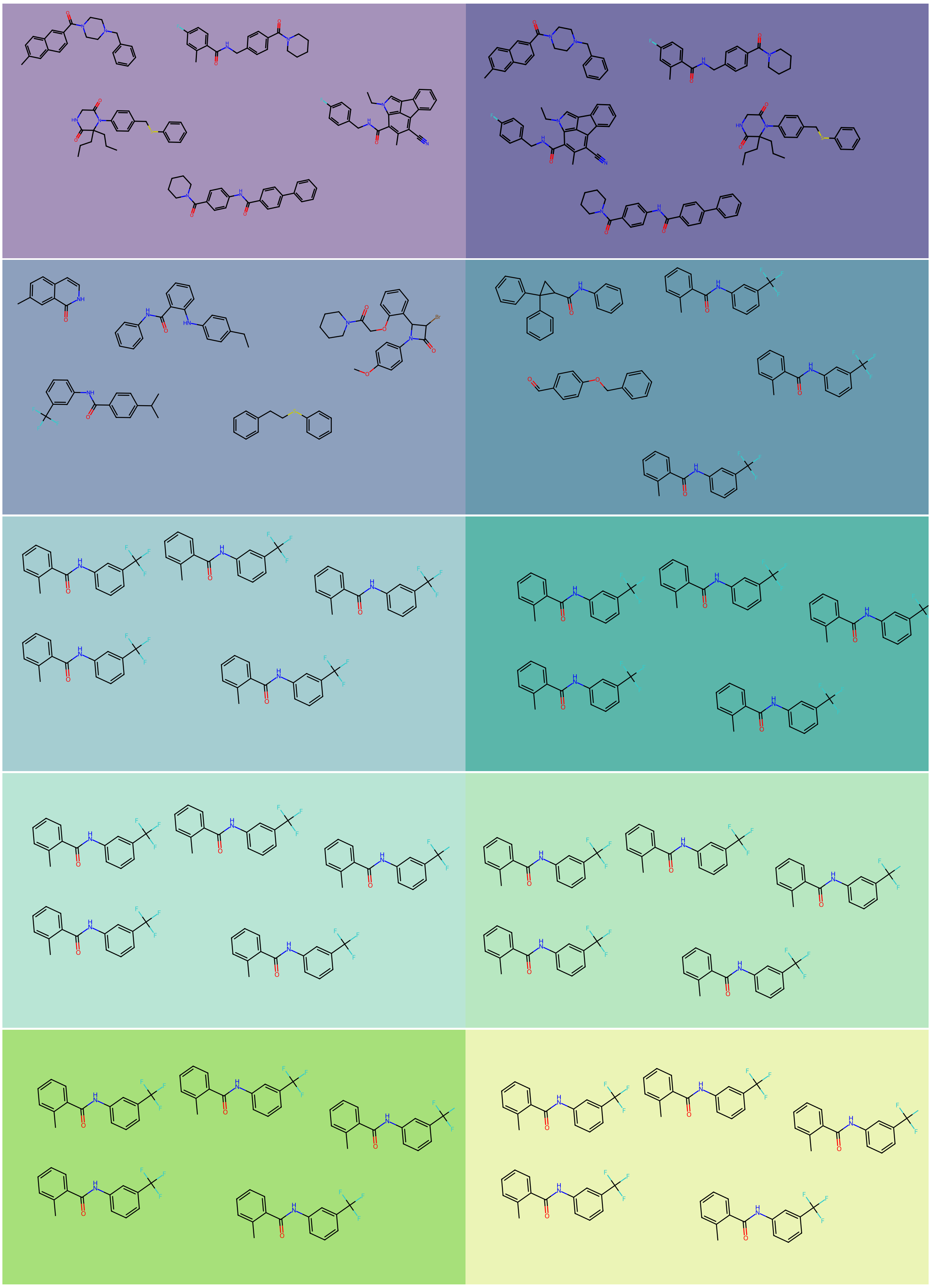

Figure S9 Molecules sampled from checkpoints for a agent trained with sum scalarization and static weight assignment to optimize for target 2. Epochs are ascending from left to right, top to bottom starting at epoch 150 up to 2850 in 150 intervals.

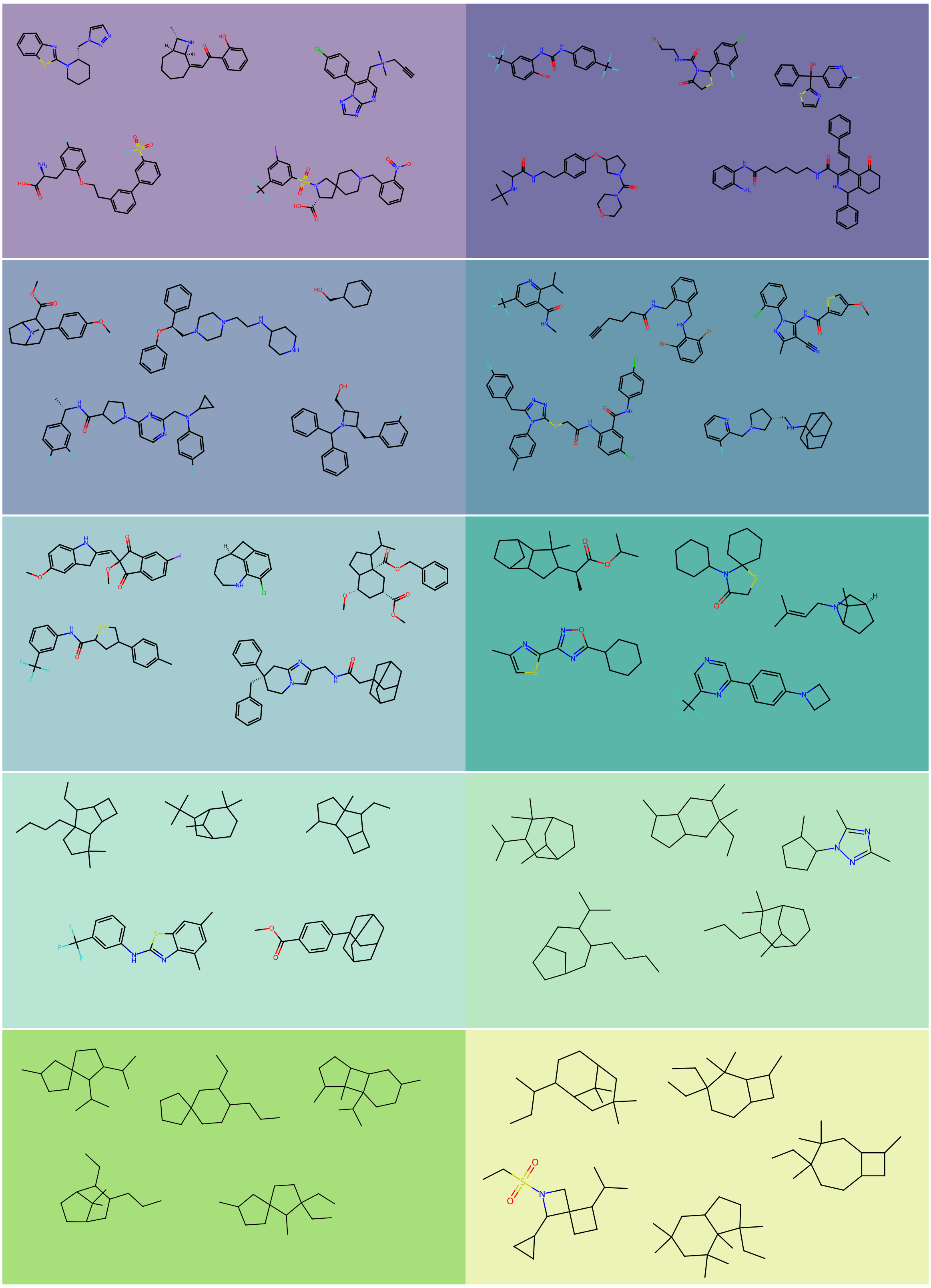

Figure S10 Molecules sampled from checkpoints for a agent trained with product scalarization and static weight assignment to optimize for target 2. Epochs are ascending from left to right, top to bottom starting at epoch 150 up to 2850 in 150 intervals.

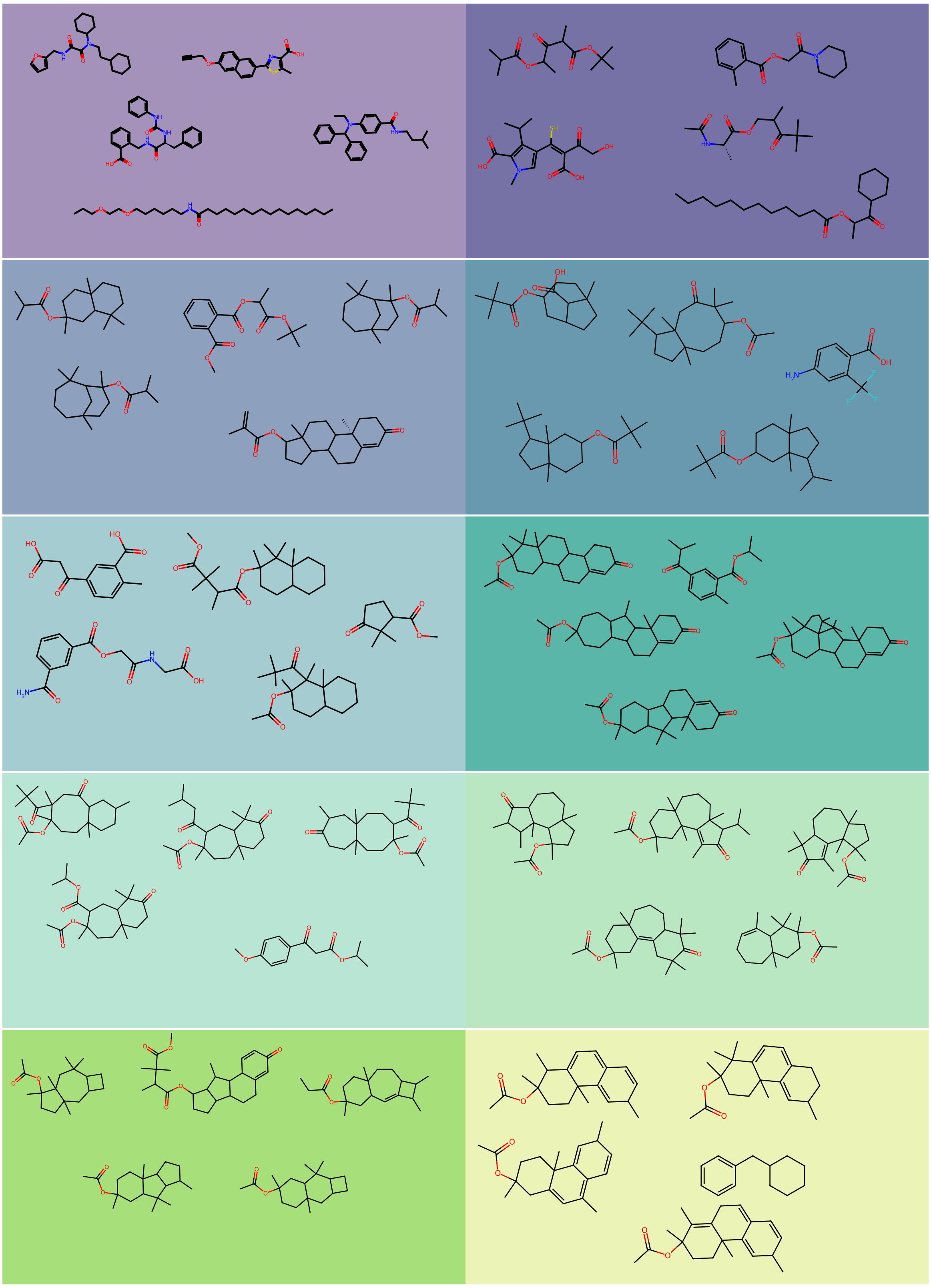

Figure S11 Molecules sampled from checkpoints for a agent trained with Chebyshev scalarization and static weight assignment to optimize for target 2. Epochs are ascending from left to right, top to bottom starting at epoch 150 up to 2850 in 150 intervals.

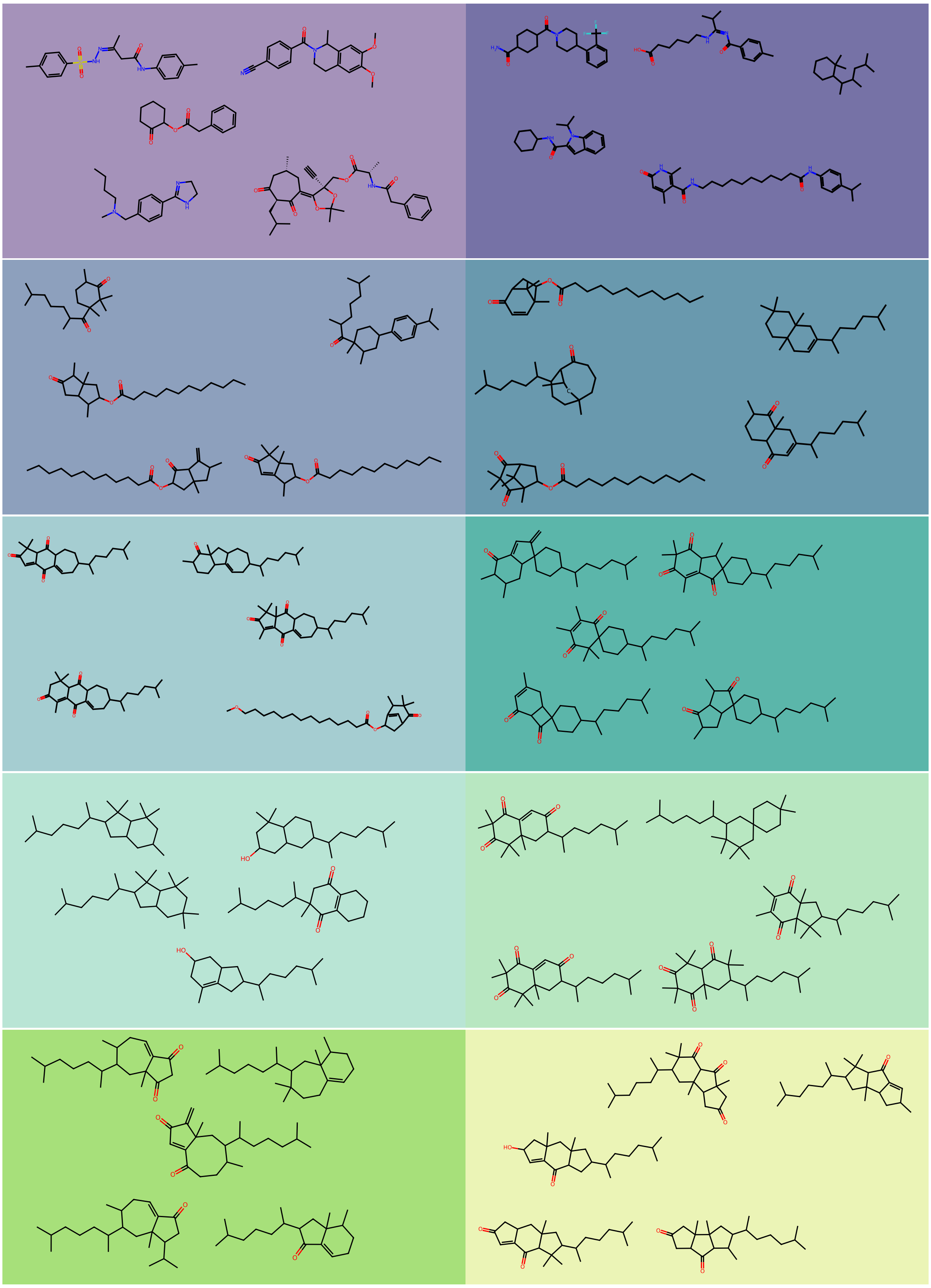

Figure S12 Molecules sampled from checkpoints for a agent trained with sum scalarization and static weight assignment to optimize for target 3. Epochs are ascending from left to right, top to bottom starting at epoch 150 up to 2850 in 150 intervals.

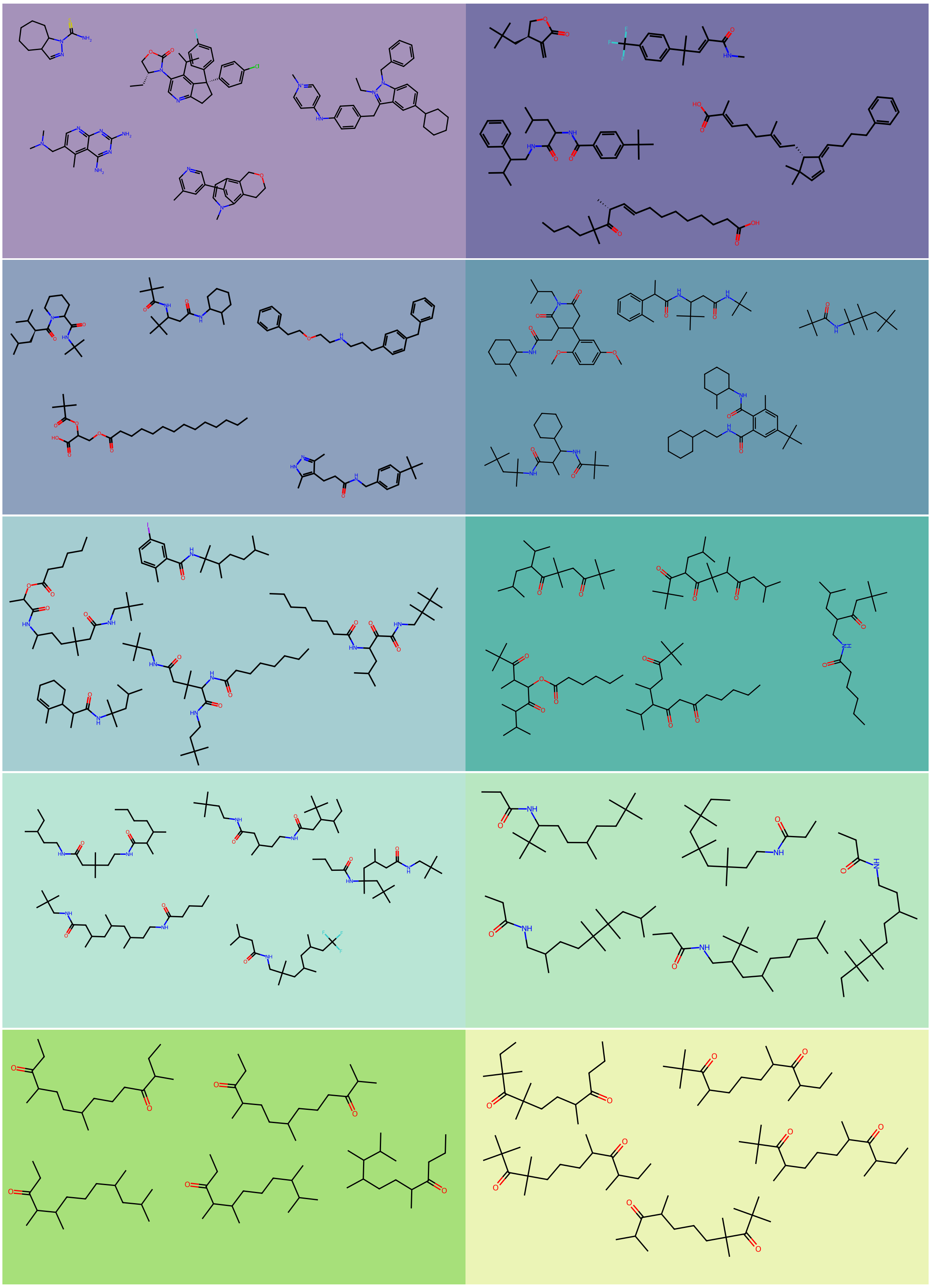

Figure S13 Molecules sampled from checkpoints for a agent trained with product scalarization and static weight assignment to optimize for target 3. Epochs are ascending from left to right, top to bottom starting at epoch 150 up to 2850 in 150 intervals.

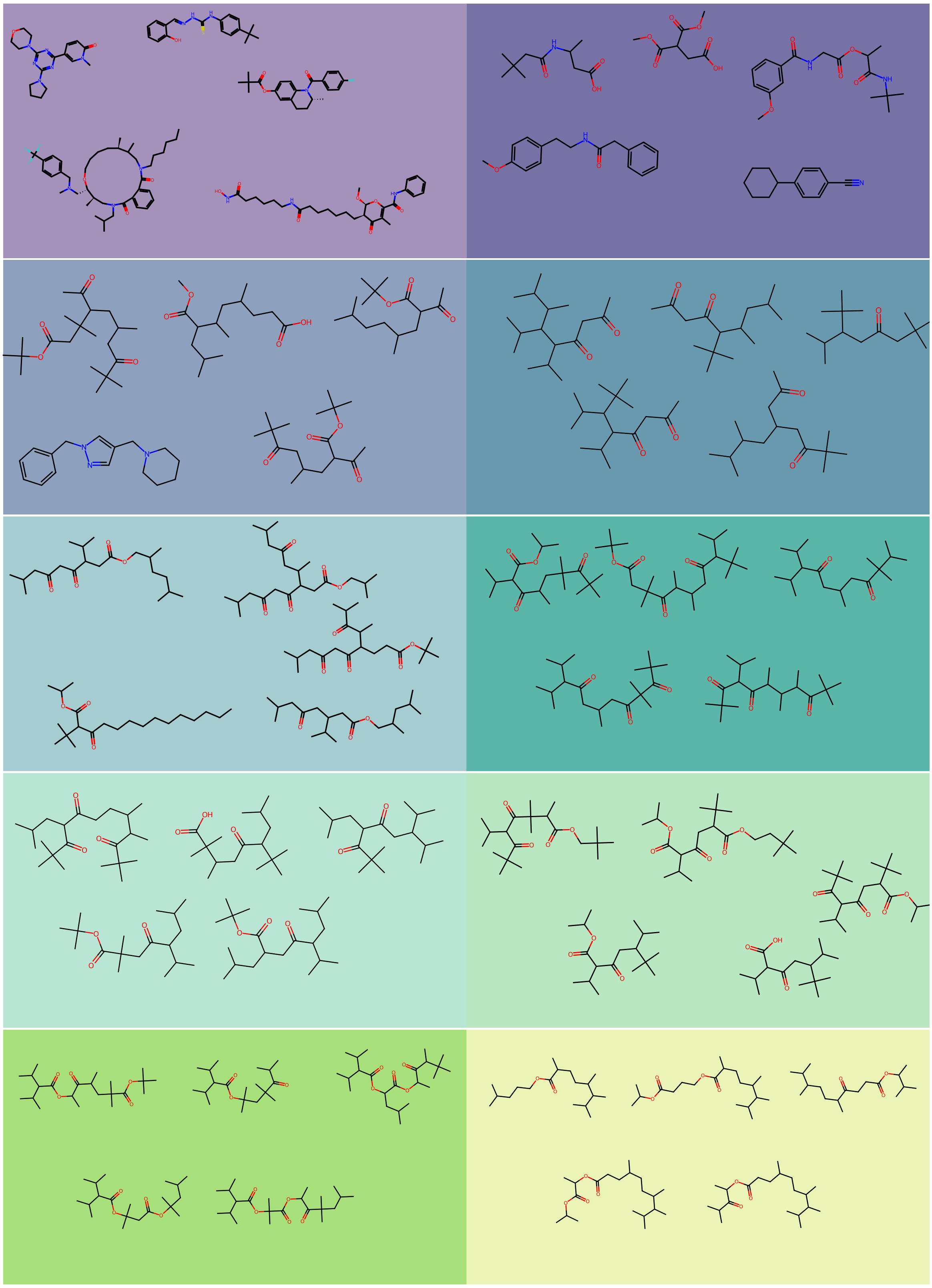

Figure S14 Molecules sampled from checkpoints for a agent trained with Chebyshev scalarization and static weight assignment to optimize for target 3. Epochs are ascending from left to right, top to bottom starting at epoch 150 up to 2850 in 150 intervals.

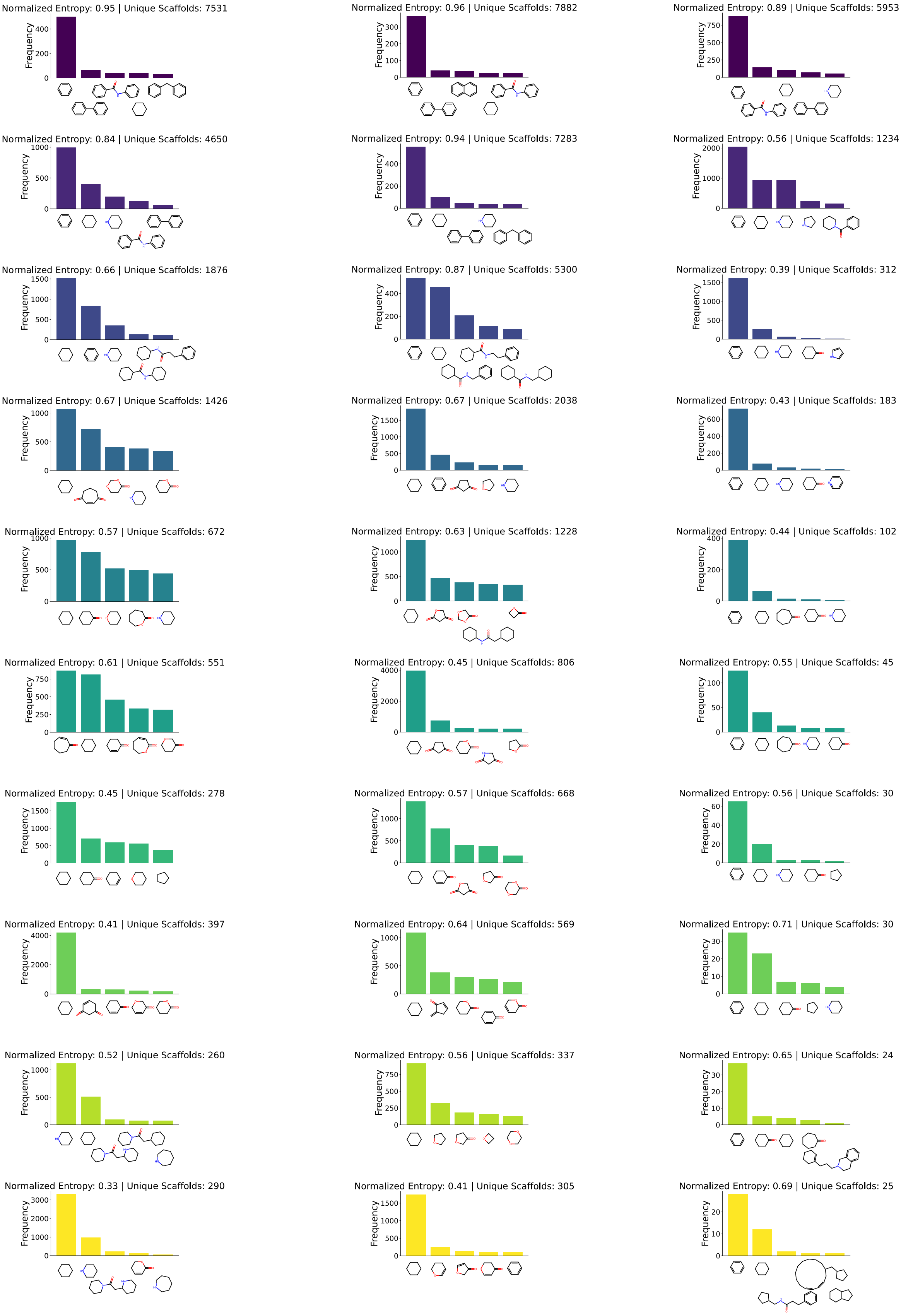

Figure S15 Top 5 scaffolds for agents trained with sum (left), product (middle) and Chebyshev (right) scalarization and static weight assignment to optimize for target 1. From top to bottom epochs are ascending, starting at epoch 150 up to 2850 in 150 intervals.

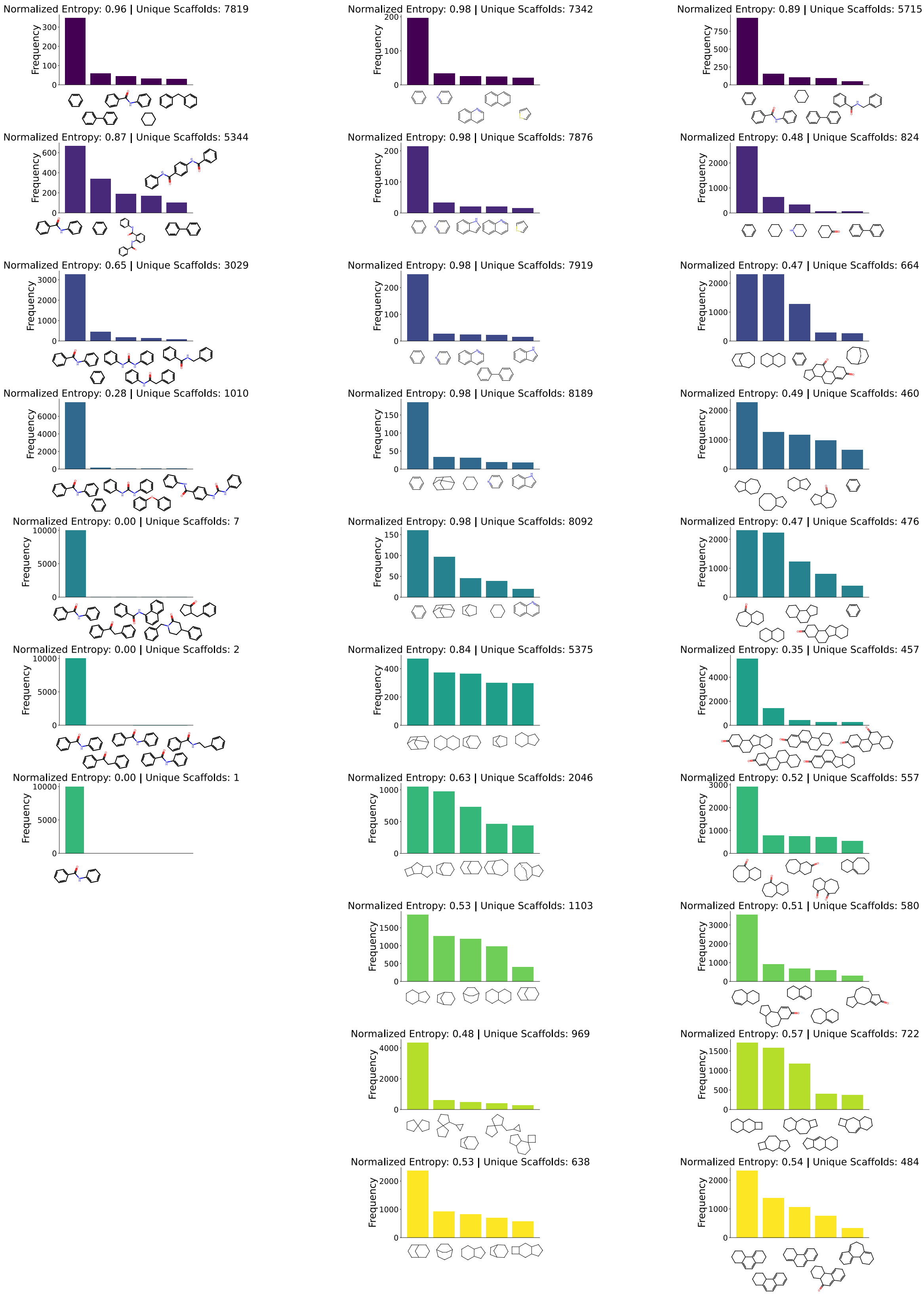

Figure S16 Top 5 scaffolds for agents trained with sum (left), product (middle) and Chebyshev (right) scalarization and static weight assignment to optimize for target 2. From top to bottom epochs are ascending, starting at epoch 150 up to 2850 in 150 intervals.

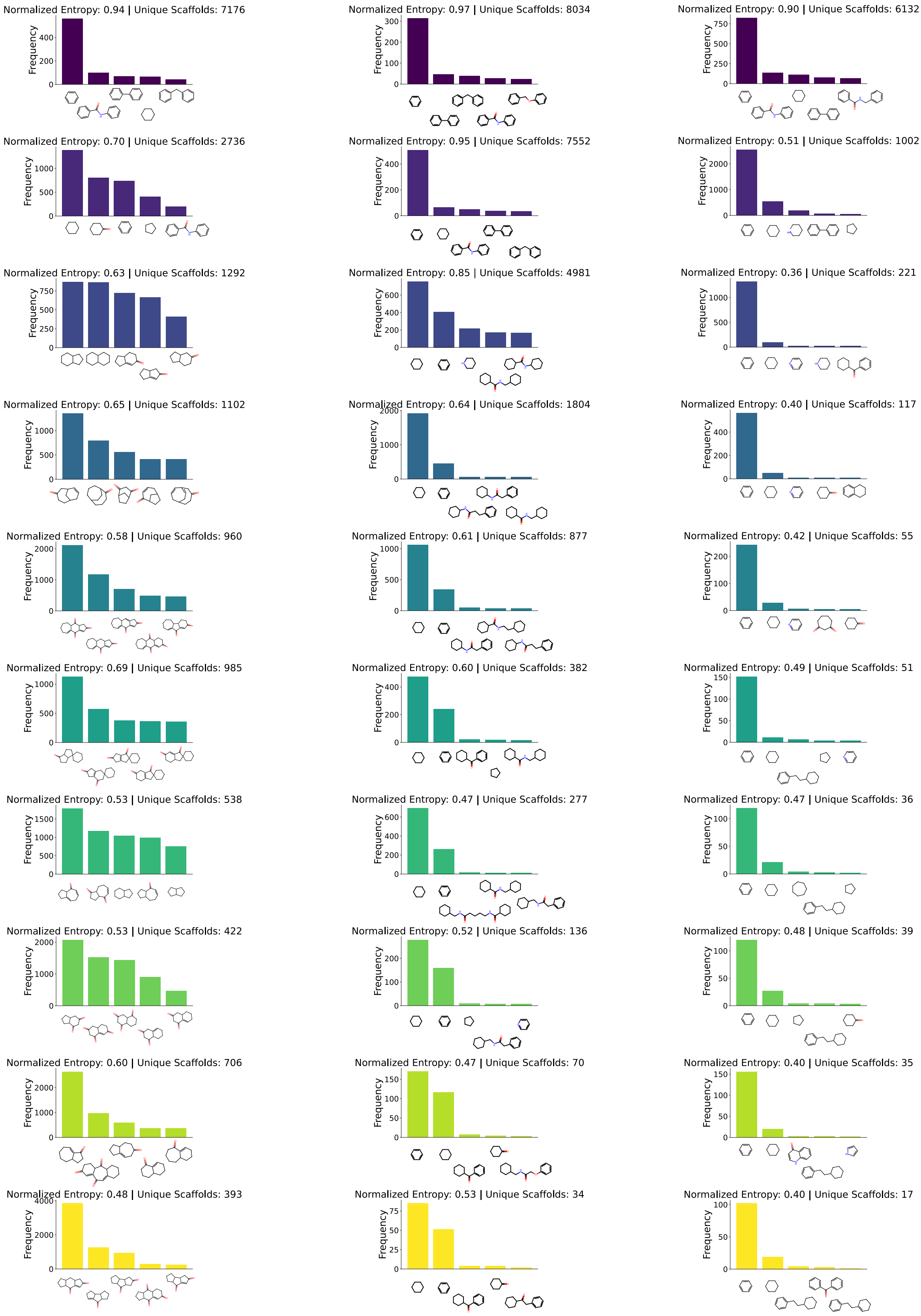

Figure S17 Top 5 scaffolds for agents trained with sum (left), product (middle) and Chebyshev (right) scalarization and static weight assignment to optimize for target 3. From top to bottom epochs are ascending, starting at epoch 150 up to 2850 in 150 intervals.

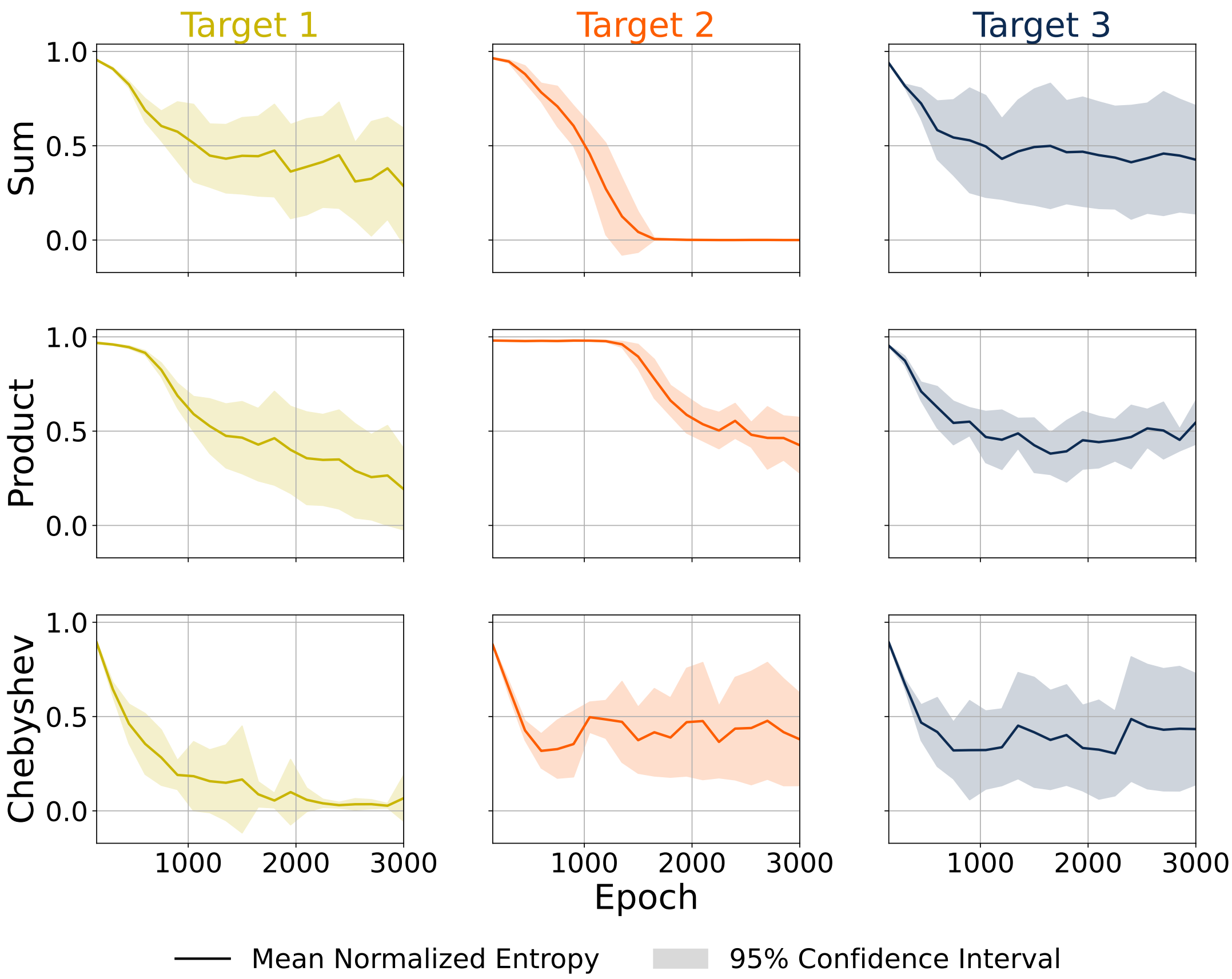

Figure S18 Evolution of NE of Bemis-Murcko scaffolds during RL fine-tuning with 100k samples for each checkpoint. Rows correspond to scalarization methods (Sum, Product, Chebyshev) and columns to target regions. Shaded areas represent the 95% confidence interval.
